# Extreme Biomimetics: Achievements and Fundamental Challenges for the Future

**DOI:** 10.3390/biomimetics11080580

**Published:** 2026-08-13

**Authors:** Hermann Ehrlich, Teofil Jesionowski

**Affiliations:** 1 Center for Advanced Technologies, Adam Mickiewicz University, Uniwersytetu Poznańskiego 10, 61614 Poznan, Poland; 2 Institute of Chemical Technology and Engineering, Faculty of Chemical Technology, Poznan University of Technology, Berdychowo 4, 61131 Poznan, Poland

**Keywords:** extreme biomimetics, structural biomimetics, functional biomimetics, biomaterials, biopolymers, 3D scaffolds, carbonization, metallization, bioinspired materials science

## Abstract

Extreme biomimetics represents a novel multidisciplinary direction within classical biomimetics and bioinspired materials science that draws inspiration from extremophiles and extreme natural habitats to create unusual approaches for the design of next-generation materials, including composites never reported or predicted before. This review is divided into several sections covering achievements and fundamental challenges in the following fields: sources of inspiration—organisms and locations; biological materials for extreme biomimetics based on examples of biosilica, cellulose, chitin, and structural proteins (collagen, byssus, silk, keratin). Additionally, conchixes of molluscan shell origin, fish scales, and spongin as a skeletal biocomposite of industrial sponge origin are represented and discussed from the viewpoint of extreme biomimetics. Finally, scientifically based but daring experimental decisions in modern extreme biomimetics, taking the examples of extreme carbonization of naturally pre-structured biomaterials, galvanobiomimetics, in-flame biomimetics, pyro-biomimetics, and ultra-high pressure mimetics are represented for the first time as current trends with challenging goals.

## 1. Introduction

A brief historical overview of the intriguing scientific field of modern biomimetics, *extreme biomimetics*, reveals that the online version of the first book entitled *Extreme Biomimetics* was published in 2016 [1]. The book consists of ten chapters in which the authors consider extreme biomimetics in terms of biomaterials-inspired chemistry, including the following aspects: thermophilic and psychrophilic biomineralization, hydrothermal and solvothermal chemistry of metal oxides and nanostructured composites, and structural bio-sculpturing and bioinspiration as tools for the design of innovative 3D materials. It is notable that even before the publication of this book, we had published in 2013–2015 several scientific articles [2,3,4,5,6,7] in which the term extreme biomimetics already appeared. However, the origins of the term *extreme biomimetics* stem from the persistence of a reviewer, still unknown to us, who, starting in 2011, did not allow our work, which finally appeared only in 2013 in *Scientific Reports* under the title “Discovery of 505–million-year old chitin in the basal demosponge *Vauxia gracilenta*” [8], to be published. According to the reviewer, the chitin we discovered in the uniquely fossilized marine sponge skeleton could not have survived the 260 °C geothermal heat known for its discovery site in Burgess Shale, Canada. It was this skeptical remark that prompted us to conduct experiments to study the thermal stability of chitin matrices isolated from modern marine sponges, which unequivocally demonstrated the thermal stability of chitin up to 400 °C. The convincing results overcame the reviewer’s resistance and the article was published, and we came up with the idea of using this property of chitin in hydrothermal synthesis reactions as a heat-resistant biological scaffold [5]. This is how extreme biomimetics was born, opening the door for numerous tests of diverse biopolymers and structured biomaterials for their chemical and physical stability and strength under experimental conditions that are extreme in the human sense. Since then, selected biomaterials have been tested in highly concentrated metal ion solutions [9], chemically and electrochemically metallized [10,11,12,13,14,15,16,17], doused with molten metal [18], plasma-bombarded with titanium [19], and carbonized at temperatures up to 2200 °C [20,21], all while maintaining their original biological architecture.

In 2022, one of our seminal articles, “The Philosophy of Extreme Biomimetics,” was published in the journal *Sustainable Materials and Technologies* [22]. In it, we formulated our own vision of the main directions of this relatively young scientific direction as follows:

- “finding corresponding natural sources and examples for inspiration;
- understanding the principles and mechanisms of biological phenomena occurring under natural extremes;
- application of already proven technologies related to the use of biological materials;
- making scientifically based but daring experimental decisions including development of new generation of composite materials.” [22].

**Figure 1:**
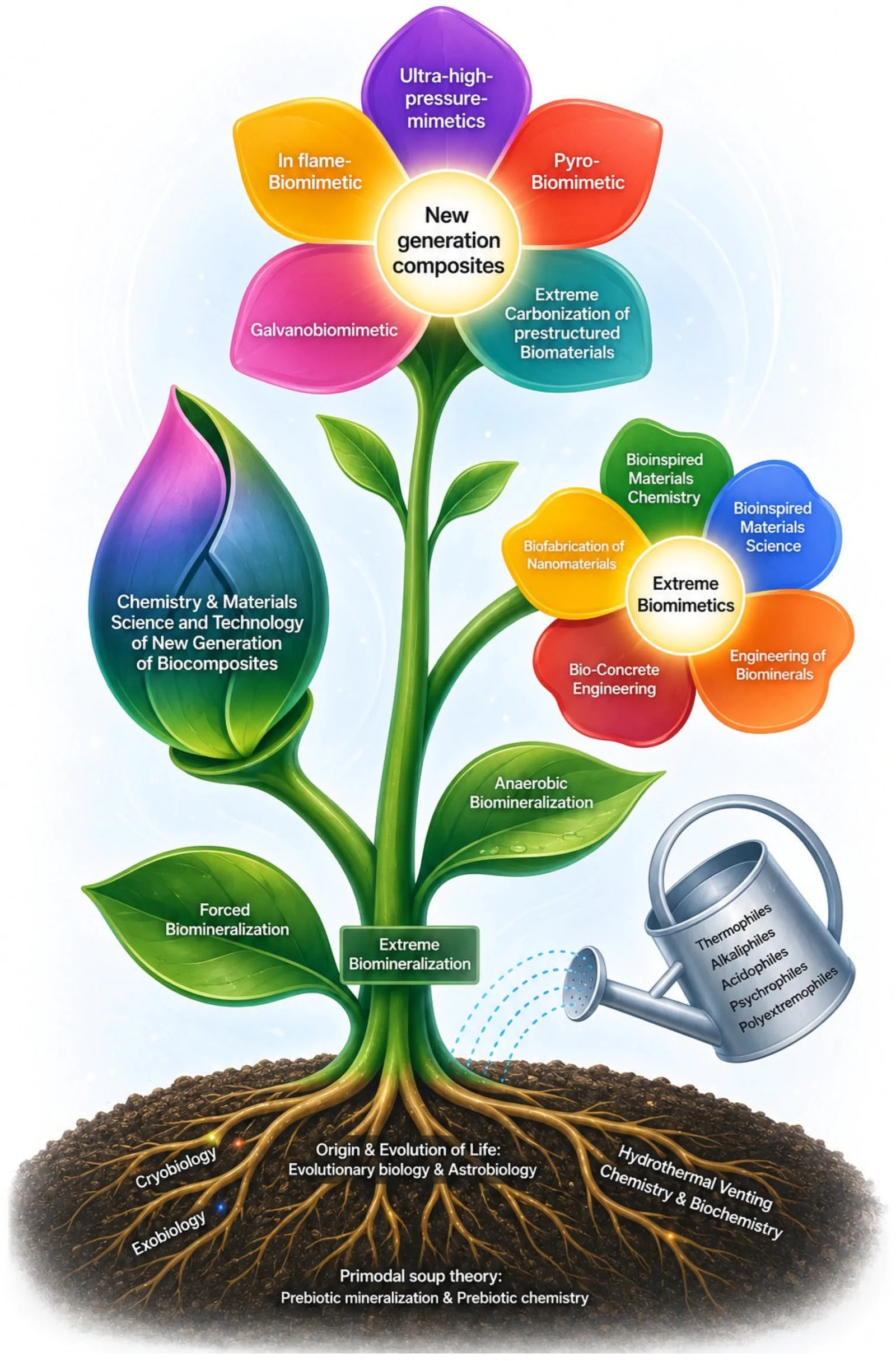
Schematic view of both extreme biomineralization and extreme biomimetics on the multidisciplinary crossroads with an outlook for the future.

In this review article, we will attempt to analyze scientific achievements and failures in the context of the above-mentioned areas and outline our vision of the prospects for the development of extreme biomimetics in the near future (Figure 1).

## 2. Extreme Biomimetics and Sources for Inspiration

### 2.1. Sources for Inspiration: Organisms and Locations

It is logical that in the search for objects of inspiration for extreme biomimetics, one should first of all pay attention to so-called extremophiles and polyextremophiles, which are represented by microorganisms. Polyextremophilic microbial communities are to be found in poly-extreme settings [23]. According to Rampelotto, such localities include, for example, “deep-sea hydrothermal vents that combine high temperature, elevated pressure, and toxic metal concentrations; hypersaline acidic lakes where saturated salinity meets low pH conditions; and high-altitude deserts marked by intense UV radiation, desiccation, and low temperature” [24]. Nowadays, evolution, molecular adaptations, diversity, distribution, and mostly biotechnological applications [25,26] of extremophiles are well represented in the literature (see for overview, [27,28,29]). However, one of the limiting factors for the actual use of such microorganisms for the purposes of modern biomaterials science is the limitation of their cultivation in laboratory conditions and the absence of macroscopic structural matrices, as is the case with the skeletons of invertebrates [30].

Invertebrates, representing around 75% of all described species on Earth, belong to 31 out of 32 animal phyla known today [31]. Numerous species, due to their evolutionary flexibility, plasticity, and variability [32], have adapted to diverse extreme environments, including those of marine origin [33]. Tardigrades are a classic example of extremophiles in invertebrates; consequently, many scientific articles have been devoted to the study of their biological resistance to extreme environmental conditions (for an overview, see [34,35]). Although the biomineralogical components of the stylet of some tardigrade species have been studied [22], the origin and nature of their claw-like structures, for example, remain unknown, although they are of interest from the point of view of biomaterials science.

Other invertebrates of interest for extreme biomimetics include marine mollusks [36], many species of which live at depths greater than 6000 m [37], or in close proximity to underwater volcanoes, in environments with high concentrations of sulfur and metal ions. For example, the world’s deepest records for two unnamed species of Neomphalidae and Sahlingia mollusks from 9577 to 9584 m, respectively, in the Kuril–Kamchatka Trench were reported in 2019 by Fukumori and others [38]. A new molluscan species in the genus *Anatoma* (Vetigastropoda: Anatomidae) has been found in abyssal hydrothermal vent field habitats on the oceanic plate margins in the Indian Ocean [39]. Of undoubted interest will also be the settling formations of biomineral nature in abyssal mollusks from regions of polymetallic ore deposits. Such species have been found in the UK–1 high polymetallic nodule exploration area, Clarion–Clipperton Zone, central Pacific Ocean [40]. The concept of forced biomineralization proposed by us earlier [41] suggests the possibility of forming various types of metal-containing biocomposite materials under conditions of local supersaturation of metal ions in the presence of organic templates. Such conditions can be created autonomously both in places of intensive extraction of polymetallic ores on the ocean floor and in hydrothermal vent areas.

It is not surprising that the objects of research in the focus of bioinspired materials chemistry turned out to include the hot-vent snail *Ifremeria nautilei* [42,43] and scaly foot snail (*Chrysomallon squamiferum*) [43]. The iron sulfide-reinforced shell skeleton of *C. squamiferum* was particularly subjected to analytical studies in depth [44,45]. Thus, according to Okada and others, this endemic snail “mediates biomineralization in its scales by supplying sulfur through channel–like columns in which reaction with iron ions diffusing inward from the surrounding vent fluid mineralizes iron sulfides” [46]. It was suggested that this biomineralogical scenario is expected to have biomimetic potential for industrial production of metal chalcogenide nanoparticles [46].

What about worms, or representatives of the phylum Nematoda and phylum Annelida? Indeed, extremophiles have been discovered and described in both nematodes [47] and annelids [48,49]. The most investigated representatives of extremophilic and extremotolerant annelids belong to such species as the vent worm *Riftia pachyptila* [50], hydrothermal vent scaleworm *Branchipolynoe symmytilida* [51,52], Pompeii worm (*Alvinella pompejana*) [53], and sulfide worms (*Paralvinella sulfincola* and *Paralvinella hessleri*) [54].

Scientific interest in *Riftia* was initially sparked by the presence of a giant tubular skeleton based on a chitin–protein complex that remains a highly interesting biocomposite [55,56]. At the same time, another deep-sea worm *B. symmytilida* is capable of existing in anoxic conditions inside mollusks and has a very specific hemoglobin [51]. The thermotolerance of the Pompeii worm *A. pompejna* [53,57] and peculiarities of its skeleton [58], including thermostable collagen [59], are still in the focus of biomaterials science. For example, the confirmed intracellular presence of arsenic and zinc associated with sulfur in this species [60] suggests the existence of corresponding unique biocomposites. Recently, an intriguing biomimetic model inspired by the Pompeii worm to reverse overheating in nanosatellites has been proposed [61]. According to the authors, “the Pompeii worm realizes the conversion of the excess heat it is exposed to into cooling inside a multilayered tube” [61]. It was also reported [62] that this vent worm has spherocrystals of biomineralogical origin which are composed of sulfur and other metals in its digestive tract. These biominerals in the form of spherocrystals may contribute to the regulation of sulfur in body fluids of *A. pompejana* [62].

Such an extremophile as the sulfide worm (*P. sulfincola*) is able to synthesize mucous tubes near hydrothermal vents and correspondingly can withstand high sulfide concentrations and temperatures [63,64]. Mucus secretion by this worm promotes the formation of a rhombic marcasite (FeS_2_) crust on vent chimneys [65]. Also, *Paralvinella* spp. forms spherocrystals containing elemental sulfur and iron in the digestive tract [62], which may also be of interest for forced biomineralization.

Another deep-sea alvinellid worm, *P. hessleri*, not only withstands vent environment temperatures above 60 °C but is also capable of accumulating exceptionally high levels of the toxic element arsenic (>1% of wet weight), as well as tolerating elevated concentrations of hydrogen sulfide, due to previously unrecognized arsenic–sulfide biomineralization [54]. Thus, Wang and others hypothesized “that arsenic accumulates within epithelial cell granules, where it likely reacts with sulfide diffused inward from the hydrothermal vent fluid, resulting in the intracellular formation of orpiment (As_2_S_3_) minerals” [54]. Such kinds of both forced and extreme biomineralization [41] undoubtedly lie within the focus of extreme biomimetics.

Extremophile species of annelids that live in the ground have also been reported. For example, *Pontoscolex corethrurus* (Annelida, Oligochaeta, Glossoscolecidae), living in the Furnas geothermal field (Sao Miguel Island, Azores), indicates a remarkable tolerance to high soil temperature as well as elevated metal bioavailability, conditions which are lethal for the majority of terrestrial metazoans [66].

Crustaceans are also certainly among the extremophiles in Earth’s deep seas [67]. It is known that diverse extremophilic marine arthropods swarm hydrothermal chimneys (for an overview, see [68]). One such representative is the blind vent shrimp *Rimicaris exoculata* [69]. Its so-called bacteriophore setae [70] are among the poorly investigated biomaterials. Specialized biocomposite-based structures in the form of insoluble granules within the hepatopancreas of *R. exoculata* are responsible for bioaccumulation of Cu, Cd, and sulfur [71,72,73]. The extremophile ostracod crustacean *Pseudocandona movilaensis* sp. nov has been recently discovered in the sulfidic chemoautotrophic ecosystem of Movile Cave in Romania [74]. Intriguingly, some crustaceans such as the brine shrimp *Artemia franciscana* have been used as a model for astrobiological studies [75].

Also, some sessile crustaceans are attached to vent chimneys. For example, the hydrothermal vent barnacle *Vulcanolepas osheai* has adapted to withstand the high temperatures and chemical toxicity of its environment, being covered with calcareous plates [76,77,78].

**Figure 2:**
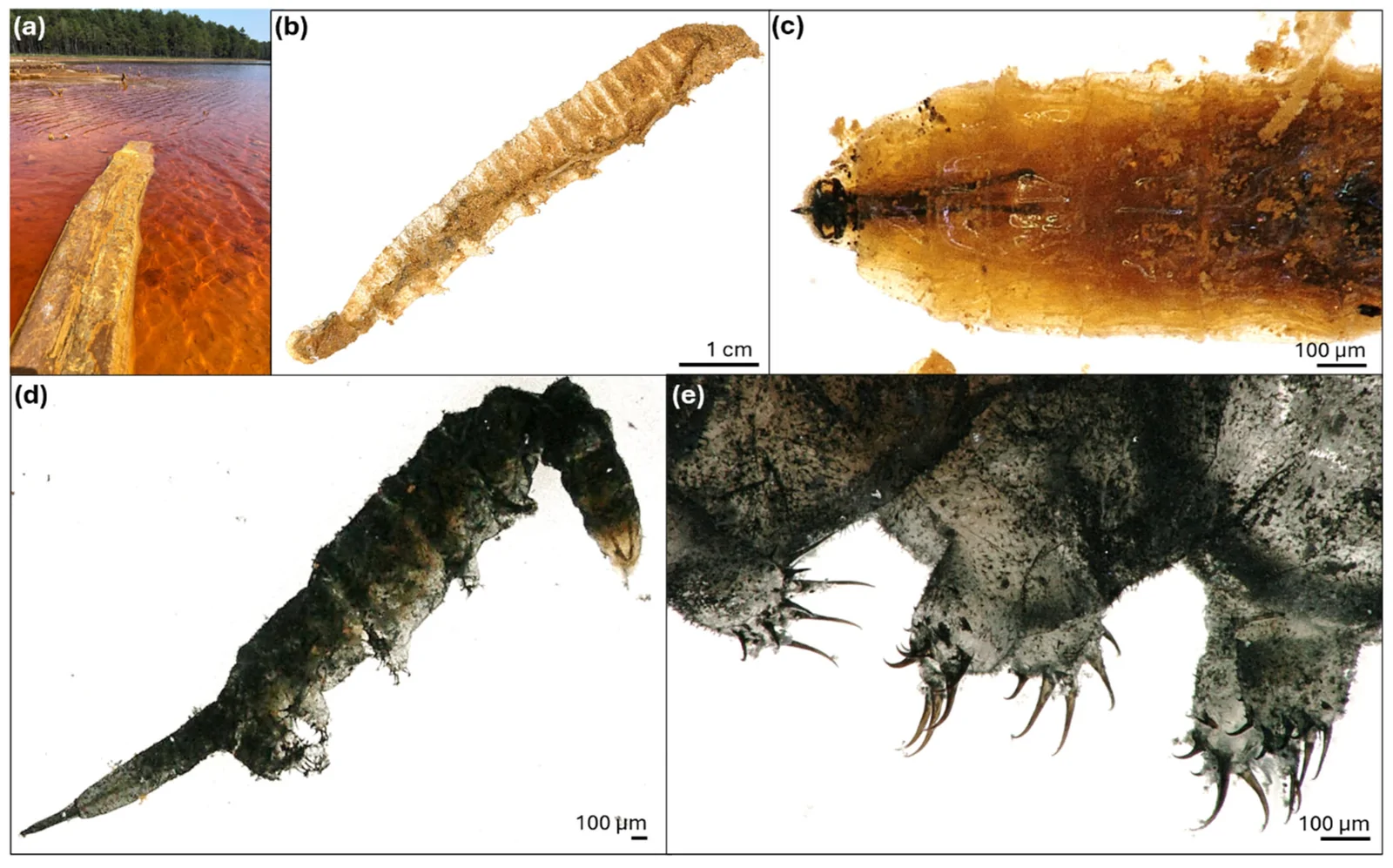
Diverse species of Corixidae can be found in an acidic lake with a pH between 3.5 and 5 near Plessa in Germany (**a**). Shore flies *Ephydra* sp. larvae (**b**,**d**) with a characteristic stylet (**b**,**c**,**e**) and claws (**e**) of unknown biomineral origin were discovered by us in mineral water sources saturated with iron, silicate, and radon gas (**b**) as well as in the mirabilite brine (**d**) in Western Ukraine.

It is precisely such representatives of arthropods as insects, or rather the larvae of some of them, that allow us to rise from the ocean depths and continue our bioinspired search in “extreme lakes.” Such lakes exist everywhere in places where various metal ores or brown coal were previously mined and processed (Figure 2a). Thus, diverse species of Corixidae (Hemiptera, Heteroptera) and their larvae have been discovered in acidic mining lakes with pH ≤ 3 in Lusatia, Germany [79]. No less exotic inhabitants were discovered by us during the expedition in July 2025 in the Carpathian region of Western Ukraine (for details, see Figure 2b–e). Such inhabitants are of scientific interest to biomaterials scientists and experts in extreme biomimetics, specifically for the possible discovery of previously unknown to science biocomposite materials with a specific structural organization at the nanoscale and a complex and possibly unexpected chemical origin.

Due to the extensive literature [80,81] on the topic discussed in this subsection, we recommend that readers familiarize themselves with a number of reviews that address other specific aspects of extremophile life, including anhydrobiotic [82], anoxic [83], and magnetic [84], as well as low temperatures [85] and radiation stress [86]. Selected properties of biological materials discovered in extremophilic invertebrates are summarized in Table 1.

It is logical to assume that unusual biocomposite structures will also form in areas of man-made catastrophic historical industrial disasters, long-standing toxic waste ponds, and, unfortunately, in areas where military operations are taking place today [87]. It is highly probable that uranium-saturated biosilicates are formed in diatoms or in sponge spicules from the Chernobyl or Fukushima zones. Biocomposite materials, which we consider exotic, can be found in organisms that have survived and are still living in regions where high concentrations of mercury and its compounds [88] are present, for example, as a result of gold mining [89,90,91].

Finally, it is also worth mentioning the specific direction of biomimetics under simulated Martian conditions, including the concept of Mars terraforming [92]. Even corresponding perspectives on exploiting biomineralization for Martian construction have been recently proposed and discussed [93]. Pillaca Llanos and others, being inspired by organisms adapted to low-temperature and high-pressure environments, proposed a methodology that includes “an in-depth review of these organisms’ biological adaptations and how they can inform robot design, the identification of relevant biomimetic principles, and an assessment of planetary conditions on moons and exoplanets with subsurface oceans” [94].

### 2.2. Biological Materials for Extreme Biomimetics

There is no doubt that materials of biological origin that can find application in extreme biomimetics must have the appropriate physicochemical and material properties. We have chosen the most well-known examples, including biosilica, cellulose, chitin, collagen, and other structural proteins.

#### 2.2.1. Biosilica

Biosilica of both diatom and sponge origin is of interest in extreme biomimetics as a specifically structured, acid-resistant, and up to 1200 °C heat-stable renewable biomaterial. As recognized in selected modern reviews, diatom biosilica in the form of microporous 3D constructs possesses thermal endurance [95], variable surface chemistry, and ability to integrate metal ions [96], including applications in lithium–ion battery anodes [97,98,99,100] and as an emerging biomaterial for energy conversion and storage [101]. Diatoms have been reported as aluminosilicate synthesis microreactors for future catalyst production [102,103]. Metallization of diatom biosilica, both as diatomite particles or cell frustules, can trigger plasmonic effects useful for the creation of high-sensitivity detection platforms, including effective nanosensors [104].

Metallization of diatom siliceous frustules can be achieved using diverse approaches which belong to extreme biomimetics. Thus, Dy-doped Y_2_O_3_-coated biosilica nanostructures with variable Dy^3+^ contents were synthesized by a facile hydrothermal technique [105]. Zglobicka and others used such current-assisted powder metallurgy techniques as pulse plasma sintering for the development of Mg–biosilica composites using siliceous cells of the freshwater diatom *Didymosphenia geminata* [106].

Modification of diatom biosilica with Ce–Tb mixed oxide twinning nanoparticles and with polyphases of quasi-crystalline Tb oxide nanoparticles has been recently reported by Wojtczak and others [107].

Also in the form of biochar obtained after anoxic pyrolysis of diatoms, their biosilica found application as a novel adsorbent for Au(III) in both synthetic and real electroplating wastewaters. This diatom biochar was found to have a high adsorption capacity of 443.0 mg/g and a superior recovery efficiency of up to 92.1% [108].

Biosilica in sponges differs from diatoms primarily in its size, which can reach 3 m in length [109,110], as well as in the wide variety of morphotypes of its spicules and skeletal matrices, from the micrometer to centimeter range (see for overview, [110,111,112]). According to the modern view, the structural diversity of sponge biosilica, including highly sophisticated honeycomb structures [113], is patterned by so-called silactins (F-actins) [110,114,115].

The biomimetic potential of sponge biosilica has been recently presented in detail in the chapter by Hermann Ehrlich entitled “Architectured Biosilica in Sponges a Unique Source for Bioinspired Design” [112]. Unfortunately, unlike diatom biosilica, the same biomaterial of poriferan origin has not yet been studied for use in extreme biomimetics, especially in terms of its metallization and participation in hydrothermal synthesis reactions.

#### 2.2.2. Cellulose

Along with other polysaccharides, cellulose, having the corresponding physicochemical and material properties, may also be of interest for extreme biomimetics. These properties include stability against temperatures up to 200–320 °C, semi-crystalline structure, high tensile strength, insolubility in water, and chemical reactivity [116,117,118]. The functionalization of different types of cellulose through their metallization has also been described in the literature [119]. Examples include the incorporation of metals into cellulose-based gels for the creation of metallogels [120], reinforcing of plant cellulose with titanium dioxide (TiO_2_) nanoparticles [121], copper electrodeposition of leaf-derived lignocellulose scaffolds for high-performance flexible electronics [122], and obtaining of metallic wood through deep-cell-wall metallization with copper [123].

Methods of hydrothermal synthesis of metal oxides, previously used in extreme biomimetics, are also known for cellulose. Thus, hydrothermal liquefaction of cellulose with Ni, Zn, and Fe has been reported [124]. Recently, Goswami and others proposed hydrothermal synthesis of SnO_2_/cellulose nanocomposites as promising materials for optoelectronic devices, gas sensors, and photocatalysis [125]. Cellulose-assisted hydrothermal synthesis of manganese cobalt spinel oxide (MnCo_2_O_4_) [126], as well as hydrothermal deposition of copper oxide nano-leaves on never-dried bacterial cellulose [127], has also been reported.

**Figure 3:**
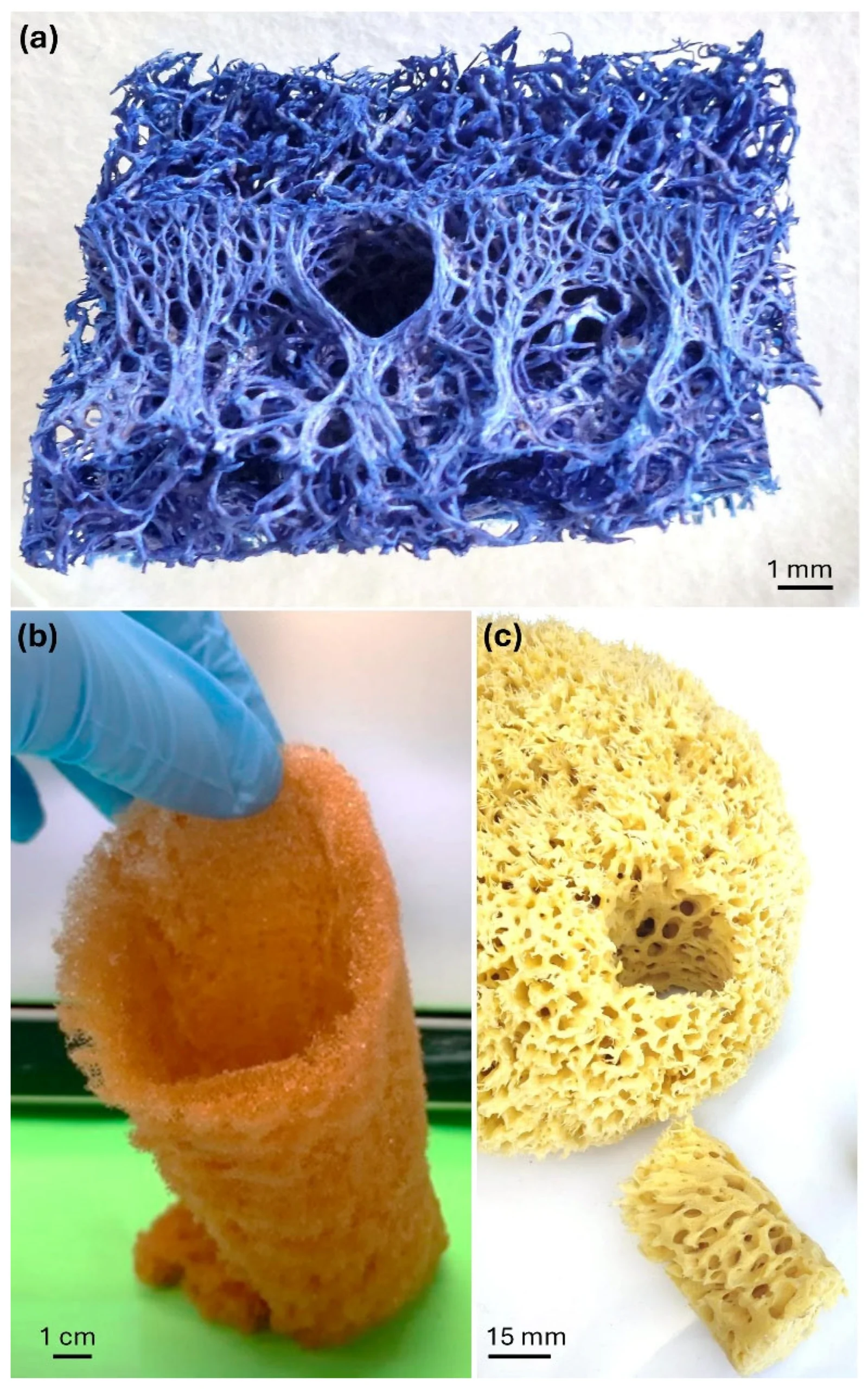
3D scaffolds used in biomaterials-inspired science and structural biomimetics. (**a**) Cellulose-based skeleton of *Luffa* sp. plant sponge colored with alcian blue. (**b**) Tube-like partially depigmented chitinous skeleton of marine demosponge *Aplysina archeri*. (**c**) Spongin-based 3D skeletal scaffold of commercial sponge *Hippospongia communis* (for details, see [19]).

A scaffolding strategy using already existing naturally pre-structured 3D scaffolds which can be isolated from renewable sources remains a trend within extreme biomimetics. The 3D skeletal construct of the cellulose-based *Luffa* plant, known also as Luffa sponge, represents an example (Figure 3a). Such features as ion transport capacity, hydrophilicity, excellent mechanical strength, adsorption performance [128], as well as sound absorption properties [129] distinguish this structure from other biopolymer-containing matrices. Specifically, the structure–function relationship of this biomaterial has inspired the fabrication of diverse biomimetic scaffolds for anti-crushing devices [130], cushioning and energy absorption [131], inorganic replicas with a very desirable size on the centimeter scale [132], and 3D-printed bionic scaffolds for bone defect regeneration [133,134].

Functionalization of Luffa sponge as a natural cellulose-based material is among the current topics (for overview, see [129]). A cobalt-based metal–organic framework (Co–MOF) on a Luffa sponge has been used for the creation of an artificial biomimetic ultra-black sponge for long-term solar-powered vapor generation and desalination [135]. Also, natural Luffa sponge-based membranes modified with metal oxides and MXenes have been used for desalination application [136]. Both high photothermal conversion efficiency and rapid water transport capability have been reported for porous structures derived from a natural loofah sponge modified with a molybdenum disulfide (MoS_2_)/reduced graphene oxide (rGO) composite for solar interfacial evaporation [137].

Carbonization of the Luffa plant sponge while preserving its primary 3D microporous structure for the purpose of subsequent functionalization also belongs to one of the areas of extreme biomimetics. This applies both to the carbonization of the Luffa sponge to obtain activated carbon for further application in supercapacitors [138] as well as to its graphitization without preliminary alkali treatment [139], however, with further chemical modification [140].

Ren and others reported the design of a hybrid 3D carbonized Luffa sponge and graphene layers encapsulated with Cu nanoparticles synthesized via a facile pyrolysis method [141]. Electromagnetic shielding material together with high flexural strength and good thermal insulation properties has been developed using a strong and heat-resistant SiC-coated carbonized cellulose-based Luffa sponge [142], as well as in the form of porous nano-Ni/Graphene/Loofah composites [143]. Carbonized Luffa plant sponge biochar, correspondingly modified with HNO_3_/NH_3,_ enabled high-performance and sustainable anodes for microbial fuel cells [144]. Three-dimensional Luffa sponge constructs continue to demonstrate a sustainable choice for highly active carbon electrodes toward supercapacitors [145]. Recently, Yan and others, with the aim of enhancing supercapacitor capacity and stability, developed a composite material designated as Ni(HCO_3_)_2_@LC–X–T using deposition of nanoscale nickel bicarbonate (Ni(HCO_3_)_2_) particles onto the Luffa-derived carbon surface with the help of a homogeneous hydrothermal precipitation technique [146]. Undoubtedly, the use of this plant sponge for the purposes of bioinspired materials science and a wide variety of areas of modern biomimetics [147,148] will continue.

#### 2.2.3. Chitin

One of the oldest aminopolysaccharides, chitin is formed when *N*-acetylated glucosamine (2-acetamido-2-deoxy-β-d-glucopyranose; GlcNAc) monomers are linked by β-1,4 glycosidic bonds [149]. Traditionally, all modern reviews of chitin indicate that it is the second most abundant polysaccharide biopolymer on the planet after cellulose, to which it is structurally analogous, with an acetamide group at the C-2 position in place of the hydroxyl group. However, we have a rhetorical question: who has currently carried out a comparative assessment between cellulose and chitin, taking into account the latest data?

After the discovery of chitin in 2009 inside the frustules of diatoms [150] that form a gigantic biomass of 0.5 gigatons [151] in the world’s oceans, or in the skeletons of numerous marine and freshwater sponges [152,153], not to mention the 1 billion tons of terrestrial arthropods [154,155], the 550 million kilograms biomass of mosquitoes [156] or 379 million tons of Antarctic krill [157], or about 300 megatons of chitin-based mycelial fungi across the planet, the time for such a recalculation is clearly ripe.

Both natural insolubility and high crystalline structure belong to the main limiting factors in chitin applications, including on the large scale. Judging only by the relevant review articles on chitin already published this year [158,159,160,161,162], this issue, along with new methods for functionalizing chitin [163], remains relevant. For example, flexible and exceptional hemostatic chitin sponges with a controllable structure have been developed using tert-butanol pretreatment [164].

In principle, the carbonization of chitin of various origins and the production of new carbonized materials still take place [165,166,167,168]. It was reported that the addition of FeCl_3_ together with mechanical activation during the pretreatment of chitin enhanced the degree of its hydrothermal carbonization. Fe^3+^ entered into the molecular chain, resulting in the destruction of the stable structure of chitin [169]. For the first time, the creation of a 3D chitin/chitosan composite scaffold from a naturally pre-structured verongiid sponge skeleton with the following metallization with Cu^2+^ ions for catalytic aims has been recently reported, too [170]. In Eike Brunner’s group, it was shown that the incorporation of graphene oxide into the chitin-based composite enhanced the inherent properties of chitin (for details, see [171,172]).

Historically, chitin has actively competed with cellulose in terms of priority for its use in extreme biomimetics. After all, the first publication on this topic in 2013 had the title “*Extreme Biomimetics: formation of zirconium dioxide nanophase using chitinous scaffolds under hydrothermal conditions*” [173]. Since then, chitin, and especially sponge-derived chitin in the form of ready-to-use cm large 3D porous scaffolds of both flat and cylindrical forms [153,174,175], has found versatile applications in extreme biomimetics and catalysis (for overview, see [2,3,4,5,6,10,11,176]) also due to its thermostability up to 400 °C [177].

What are the future prospects for using chitin for extreme biomimetics? We believe the use of ready-made chitin matrices, similar to 3D sponge scaffolds, could be of interest. Tarantula cuticles, up to a million of which are discarded as waste each year by hobbyists, could be used as a potential raw material [178,179]. These microtubular formations are made of a chitin–melanin biocomposite. Incidentally, similar composites of chitin with diverse naturally occurring pigments represent separate templates for extreme biomimetics. For example, a bioinspired dopamine coating procedure was adopted to intercalate high-content silver nanoparticles into the chitin nanocrystal matrix for the creation of corresponding chitin membranes [180]. Machalowski and others developed composite material in the form of bromotyrosine-containing 3D chitin-based skeletal scaffolds of poriferan origin covered with silver nanoparticles (AgNPs) and Ag–bromide [177]. Although the extraction of brominated chitin matrices from cultivated marine verongiid sponges is a one-step process involving only alkali treatment [181,182], these unique naturally halogenated 3D scaffolds have not yet found application in bioinspired materials science.

We deliberately did not discuss chitosan in this subsection as an extremely popular derivative of chitin, due to the fact that it is not a natural biopolymer, but is obtained as a result of chitin deacetylation.

#### 2.2.4. Structural Proteins: Collagen, Byssus, Silk, Keratin

Such structural proteins as collagen, byssus, silk, and keratin play a fundamental role in shaping the skeletons of organisms as well as corresponding structures of cells and tissues [183]. Consequently, the design of these highest-performance materials in nature [184] due to self-assembly and sustainability of polypeptide-containing biomaterial systems remains a focus of bioinspired materials science and biomimetics [185]. Many of the mentioned structural proteins are part of industrial waste of biological origin and, accordingly, are subject to effective disposal or modification into new products that are useful for society and safe for the environment.

##### Collagen

Collagens, which arose in the first multicellular organisms like sponges back in the Precambrian [186,187], are, with 28 members of the collagen family [188], the most abundant structural proteins in all vertebrates, including mammals. Some of them act as raw materials for the production of various types of biomaterials and composites, mainly for biomedicine (for overview, see [189,190,191]). Over the past decade, there has been a shift towards focusing scientific and industrial interests specifically on collagens of marine origin [192,193,194,195,196,197,198]. Of particular interest is the extraction, processing, and use of collagen from jellyfish [192,199,200,201]. However, of greater industrial significance is collagen extracted from fish scales, including those from commercial marine fish (for details, see [202,203,204,205,206,207,208]).

Only those varieties of the above-mentioned collagens of different origins that have increased thermal stability and the ability to be effectively modified with metal ions will undoubtedly find use in extreme biomimetics. So, thermostable type I collagen from the swim bladder of Silver Carp (*Hypophthalmichthys molitrix*) with a thermal denaturation temperature of 40.08 °C belongs to such examples [209]. Studies on the thermal denaturation of collagen by microthermal analysis have shown that thermal transitions identified at (150 ± 10) °C and (220 ± 10) °C can be related to the process of gelatinization of the collagen fibrils [210]. However, such reaction conditions do not exclude the possibility of using collagen in hydrothermal synthesis approaches.

Modification of collagens using metal ions is also known in the literature. According to Li and others, cross-linking collagen with high-valent metal ions such as Cr (III), Zr (IV), Fe (III), and Al (III) significantly enhances its physicochemical properties [211]. Metal-chelated collagen extracted from pleco skin (*Pterygoplichthys pardalis*) has been used as a novel biomaterial with significantly increased mechanical properties due to its electrostatic interactions with metallic ions [212]. Incorporation of metal-doped silicate microparticles into collagen scaffolds for biomedical applications with respect to endochondral bone healing has also been recently reported [213].

In our opinion, metal tanning of the collagen matrix with Fe, Cr, Al, Ti and Zr, both individually and as part of leather compositions, is clearly related to the harsh conditions of modification of biopolymers adopted in extreme biomimetics. All of these tanning agents are able to stabilize collagen and impart varying degrees of its hydrothermal stability [214], of course, if they are used at certain concentrations.

Iron tanning has been around for about a century [215] and has competed with leather chromium plating [216]. Chromium has been recognized as an extensively used metal ion for leather tanning as it binds strongly with collagen and enhances its stability [217,218]. Mechanisms of Zn (II) bound to collagen as well as new approaches of zirconium tanning have been investigated in diverse papers [219,220]. Thus, recently, a novel Zr-based halide perovskite tanning system for effective Zr^4+^ penetration in leather collagen has been reported [221,222]. Also, aluminum is a recognized tanning reagent that is widely used in the leather industry to stabilize collagens [223,224].

One of the goals of extreme biomimetics is to study the behavior of biopolymers, including collagens, in critical situations when metal concentrations are very high. For example, in one of our recent publications, we reported the testing of collagen-containing spongin for its stability using 25% and 50% concentrations of (Cr(OH)SO_4_) [17]. We believe that “*the modelling of such a situation has important practical significance for cases of leakage of highly concentrated solutions of chromium salts, for example, during industrial accidents or military conflicts, which unfortunately occur in our time*” [17].

##### Byssus

Chemically modified collagen found in natural conditions, which is found only in Bivalvia mollusks [225] and performs an adhesive function, is known as byssus. According to the classical work by Qin and others from 1997, “*mussel byssus has collagen with silk-like domains*” [226]. Byssus threads are mainly composed of fibrillar collagens which are embedded in a proteinaceous matrix [227]. Incidentally, biotechnological production of the mussel byssus-derived collagen preColD has already been reported [228]. Recently, 309 differentially expressed proteins have been shown in byssus using liquid chromatography–tandem mass spectrometry (LC–MS/MS) [229]. The key byssal proteins are represented by collagen-like proteins (e.g., C1q domain-containing proteins), mussel foot proteins (e.g., Mfp3, Mfp6), and enzymatic regulators (superoxide dismutase, tyrosinase/TYR–C, peroxidase/BPLP–1) [229].

Due to their self-healing properties [230], remarkable hierarchical structure, and unique robustness due to the specificity of their mechanical properties [231], byssus is of biomimetic importance for the development of novel underwater bio-adhesive agents [232] as well as of diverse next-generation biomaterials and healthcare technologies [233]. It is to note that the byssus of marine bivalvian *Atrina pectinata* is to be found in the form of threads which display a brilliant golden hue arising from structural coloration, ensuring exceptional lightfastness [231].

Interaction of byssus with diverse metal ions including Ca, Mg, Fe, Cd, Al, Zn, and V is well investigated in numerous publications (for details, see [234,235,236,237,238]). Despite the fact that at present, with the flourishing of aquaculture of edible marine mollusks, byssus has become classified as mariculture waste [239], and its use in the creation of metal oxide composite materials obtained by hydrothermal synthesis methods according to the tradition of extreme biomimetics is still absent. A similar situation arose with the carbonization of byssus.

##### Silk

Structural protein silk is produced by diverse representatives of arthropods [240]. Fibroins in silkworm silk as well as spidroins in spider silk, which are characterized by repetitive sequence motifs, remain the main structural component of it (for an overview, see [241]). In addition to fibroin, silkworm silk also contains the glue-like protein sericin, which can be removed by a thermo-chemical treatment, also known as degumming [242]. Silk-like structural motifs are to be found in diverse biological materials [243], including marine tube-building corophioid amphipods, that produce from their legs fibrous, adhesive underwater threads known as “marine silk” [192,244,245].

According to Aikman and others, “*leveraging the biodiversity in arthropod silks offers advantages due to the diverse mechanical properties and thermal stabilities achievable, primarily attributed to variations in fiber crystallinity and repeating amino acid motifs.*” [246]. From an extreme biomimetics view, it is important to know that silk belongs to a highly heat-sensitive protein and begins to decompose at approximately 165 °C [247]. This property was taken into account due to the production of silk-based composites, fibers, sponges, hydrogels, tubes, microspheres, and thin films [248,249,250,251,252]. Recently, the so-called “Hydration–Crystallization Locking” strategy has been proposed with respect to silk fibroin modifications. Using this approach, a flexible fibroin membrane exhibiting toughness (∼16.4 ± 1.2 MJ/m^3^), high tensile strength (∼50.5 ± 3.2 MPa), and >95% shape retention under large deformations at −196 °C and 70 °C has been created [253].

Metallization of silk, both fibroin and sericin, is well investigated [254,255]. Treating silk with metal salts was a common practice until the early 20th century [256]. For example, tin salts, as the most commonly mentioned metal salts, have been traditionally used for weighting silks of any color [256]. Boiling silk in a metal solution involves weighting (soaking silk in metal–salt solutions to increase weight, density, and drape) or degumming (removing natural gummy proteins using alkaline solutions). It was reported that metal ion coagulation baths may make silk fibers stronger than native spider silk [257]. An injection of metal by infiltrating the protein structure of the silk makes this biopolymer more resilient. Thus, Liquid Metal@Silk Fibroin hybrids have been created recently [258]. Numerous metal oxide–silk composites including ferric oxide [259,260], zinc oxide [261], manganese oxide [262], and tin/bismuth oxide [263] have been developed in the last decade. Biomimetic nucleation of diverse metal–organic frameworks on silk fibroin has also been reported [264].

Of course, silk and its derivatives have not escaped research into their carbonization and the creation of new composite materials (see for overview, [259,265,266,267]). A highly efficient metal-free electrocatalyst for the oxygen reduction reaction has been developed using nitrogen-doped porous activated carbon [268]. Facile carbonization and chemical activation of silk fibroin by KCl led to the production of cobalt and tungsten dual metal-loaded N-doped porous carbon electrocatalysts used for the hydrogen evolution reaction and oxygen evolution reaction [269]. In situ preparation of molybdenum dioxide-incorporated carbonized silk fiber and its application in flexible supercapacitors has been carried out, too [270]. According to Ji and others, they “*sprayed ammonium molybdate tetrahydrate (AMT) on the surface of mulberry leaves used for feeding silkworms and investigated the effect of feeding AMT on the growth of silkworms and the properties of spun silk. The precursor incorporated into the silk was converted into scattered MoO_2_ NPs, which were embedded within the carbonized silk fiber via carbothermal reduction*” [270]. Hierarchically porous carbon obtained from silk cocoons has been successfully used for high-performance Li-O_2_ batteries [271,272], as well as for an anode for highly robust Na-Ion hybrid capacitors [273]. A flexible piezoresistive sensor was successfully prepared by encapsulating carbonized flat silk cocoons within an elastic matrix of polydimethylsiloxane [274]. Also, the development of silk-based pseudographitic pyroproteins has been reported [275,276]. It was shown that by simple heating to 350 °C, the β-sheet structure of silk protein can be transformed into an sp2-hybridized carbon hexagonal structure. Further heating of the pseudographitic crystalline layers to 2800 °C led to the formation of highly ordered graphitic structures [275].

What about silk as biowaste? [277]. For example, silk sericin, accounting for approximately 20–30% of the silk mass, was previously considered a waste by-product in silk fibroin extraction [278,279]. Now, sericin is recognized as a next-generation biomaterial [280,281]. It can be effectively metallized using Pb^2+^, Co^2+^, Ni^2+^, Cu^2+^, and Zn^2+^ [282]. Sericin also mediated gold/silver bimetallic nanoparticles applicable for photocatalytic degradation of waste materials [283]. It was reported that the incorporation of nanosized metals and metal oxides into sericin-based electro-spun nanofibers imparts new functions to the produced composites [284].

**Figure 4:**
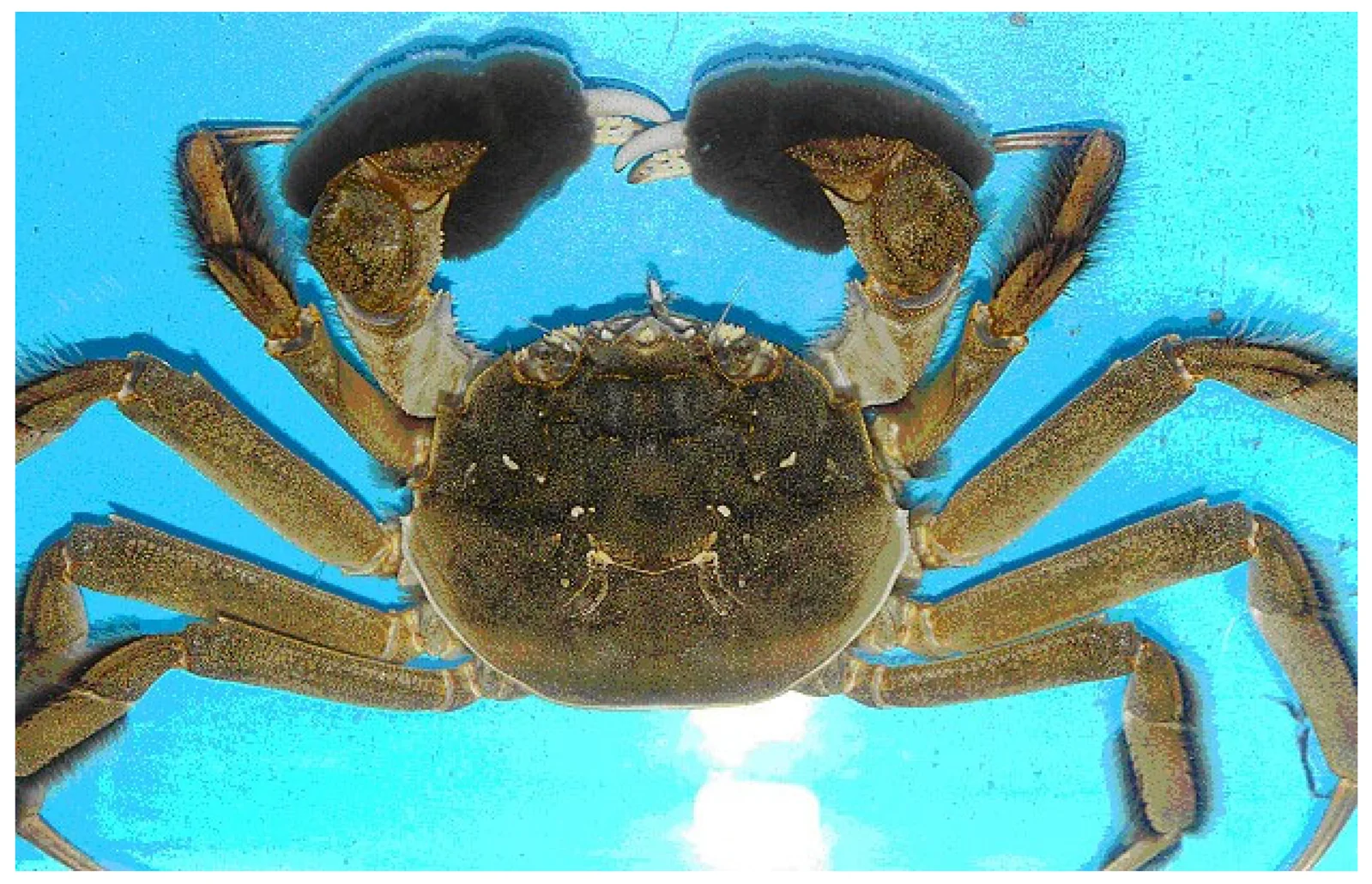
Chinese mitten crab (*Eriocheir sinensis*) possesses setae-containing fur-like formations on its claws. These setae are examples of some kind of still poorly investigated silk–chitin biocomposites.

Could other renewable raw materials for silk production be discovered for the purposes of extreme biomimetics? We suggest that the Chinese mitten crab, also known as *Eriocheir sinensis* (Figure 4), as an economically valuable species widely farmed in South-East Asia and China [260,285,286], is one such source. The claws of this crab, invasive to Europe and the United States, are densely covered with hairs called setae, which consist of silk fibers coated with a nanolayer of chitin, as discovered by us previously [192]. Unfortunately, this unique silk-containing biomaterial has remained virtually unstudied, although it is available in large quantities due to its classification as marine biowaste.

We have no doubt that silk will provide researchers with new surprises that will also be relevant to some aspects of extreme biomimetics. Thus, in a 2026 paper published in *Nature Sustainability* entitled “*Hierarchical materials from fused silk*”, Zhou and others represented a thermomechanical process to fuse silk fibers into solid materials of arbitrary shapes similar to strong plastic-like materials with 6G potential [253]. These silk fibers have been fused at extreme conditions such as 311 degrees Fahrenheit and around 9800 atmospheres of pressure.

##### Keratin

Among such structural and fibrillar proteins as intermediate filament proteins, cysteine-rich (approximately 10% *w*/*w* of protein) keratins represent the largest subgroup [287,288] and are the most important biopolymers in mammals after collagen [289]. Chemistry, structure, function, and diverse features of keratins are represented in numerous comprehensive reviews (see for overview, [290,291,292]). The limiting factor in the use of keratins for extreme biomimetics is their high insolubility in water, weak acids, and organic solvents. In the fundamental paper by Woodin already published in 1954 in *Nature*, they found that “*the stability of keratin in the solid state is due to cross-linking of the polypeptide chains by hydrogen bonds, disulphide linkages and electrostatic forces, and under conditions where these bonds are broken solutions of the protein are obtained*” [293]. Accordingly, a large number of studies have been and continue to be devoted specifically to the development of methods for extracting keratins and solubilizing them for further use. The driving force of these studies is the occurrence of millions of tons of keratin-based biological materials (hairs, pig bristles, wool, feathers, hooves, horns, etc.) as biowaste [294] that must be utilized, chemically modified, functionalized, and finally used for biomedical, ecological, or bioinspired materials science aims [295], including engineering [296,297,298], production of bioplastics [299,300], 3D printing [301], or development of supercapacitors [302].

Modern methods of extraction and solubilization of keratins (for an overview, see [303,304,305]) include the application of ionic liquids [306,307,308], deep eutectic solvents [309], as well as such reagents as NaOH–urea, NaOH–thiourea, and sodium sulfide [310]. Keratins, dissolved to varying degrees, are now subject to functionalization through interaction with organic and inorganic substances, which leads to the creation of a variety of composite materials with a wide range of practical applications. Keratin associations with synthetic, biosynthetic, and natural polymers (see for review, [311]) have been reported in examples of keratin–cellulose composites [312], keratin-β-polypropylene composites [313], keratin–bioglass–polycaprolactone composites [314], and keratin–polyethylene oxide composites [315]. Created amidoxime-modified feather keratin aerogel was used for efficient (585.88 mg·g^−1^) uranium adsorption [316]. Moreover, it is functionalized keratin that effectively adsorbs metals up to 695 mg/g from aqueous solutions [317]. Also, alkaline ultrasonic treatment of the keratin fibers resulted in a multi-fold increase in metal uptake [318].

Much time has passed since the publication of Sikorski’s and Simpson’s fundamental article in 1958, entitled “*Studies of the reactivity of keratin with heavy metals*” [319]. Until now, the interaction of keratins with the formation of corresponding complexes or composites is known for such metals as Au [320,321], Ag [322,323,324], Ti [325], Cr [326,327], and Li [328]. Magnetically recyclable keratin of wool origin modified magnetite powders and was effectively used for the removal of Cu^2+^ ions from aqueous solutions [329]. As recently reported by Aleinawi and others, phosphoric acid-functionalized keratin exhibited exceptional selectivity for Li ions as well as the highest 189.8 mg/g lithium adsorption capacity [302].

Due to the existence of a number of biomineralogical scenarios in which keratin acts as a template for calcification [330,331], the topic of creating mineral–keratin composites continues to be intriguing. It was hypothesized [332] that due to their unique elasticity and toughness, hair keratins stabilize the enamel rod sheaths and contribute to the perfect balance between hardness and fracture toughness of enamel. Until today, biomimetic mineralization of selected keratin scaffolds (i.e., including via electrodeposition) [333] remains a current trend [334,335].

The development of keratin-based composites with graphene oxides (GOs) belongs to one of the modern biomimetic directions. Thus, the creation of wool keratin and GO composites [336,337], as well as of keratin/polylactic acid/GO composite nanofibers, was reported on [338].

The thermal stability of keratin, like other aforementioned biopolymers used in extreme biomimetics [339], is a key factor. It was shown that the elimination of water brings greater thermal stability of selected keratins (i.e., fowl feather keratin). Thus, in dry conditions, thermal transition of this keratin occurred in a temperature region close to 170–200 °C [340]. In a number of studies, keratins were also carbonized at different temperatures and with different end goals [341]. Activated carbon has been produced from yak hair keratin waste using a steam flash explosion technique [295]. Also, diverse keratin-derived biochars have been reported in the literature (see for overview, [342,343,344]). Attention has also been paid to keratin-derived functional carbon with superior charge storage useful for the development of high-performance supercapacitors [345,346].

An extremely interesting situation is currently developing around the hypothesis of a possible keratin origin of the nanostructured cuticular layer of spongin fibers [21], a specific biocomposite material that makes up the microporous 3D skeletons of so-called marine bath sponges (Figure 3c), also classified as commercial or industrial species [19,347]. The special role of spongin in the development of extreme biomimetics is presented below.

#### 2.2.5. Biocomposites: Conchixes, Fish Scales, Spongin

Biocomposites usually include two or more components, organic or inorganic (i.e., biominerals) in nature, but necessarily of biological origin, that is, synthesized only by biological systems. Below, we present and discuss the three selected examples of biocomposites from the view of extreme biomimetics, namely conchixes of molluscan shell origin, fish scales, and spongin as skeletal biocomposite material of some species of industrially important marine demosponges.

##### Conchixes

The shells of marine mollusks, which are industrial species and grown globally in aquaculture, are a well-known type of biological waste. However, being a new source of sustainable biomaterials [348], they also possess enormous biomimetic potential [349,350]. Molluscan shells have already found applications as a sustainable source of aragonite-rich calcium carbonate for advanced dental biomaterials [351], as eco-friendly substitutes for synthetic fillers in friction composites [352], as well as a source of calcium lactate [353].

**Figure 5:**
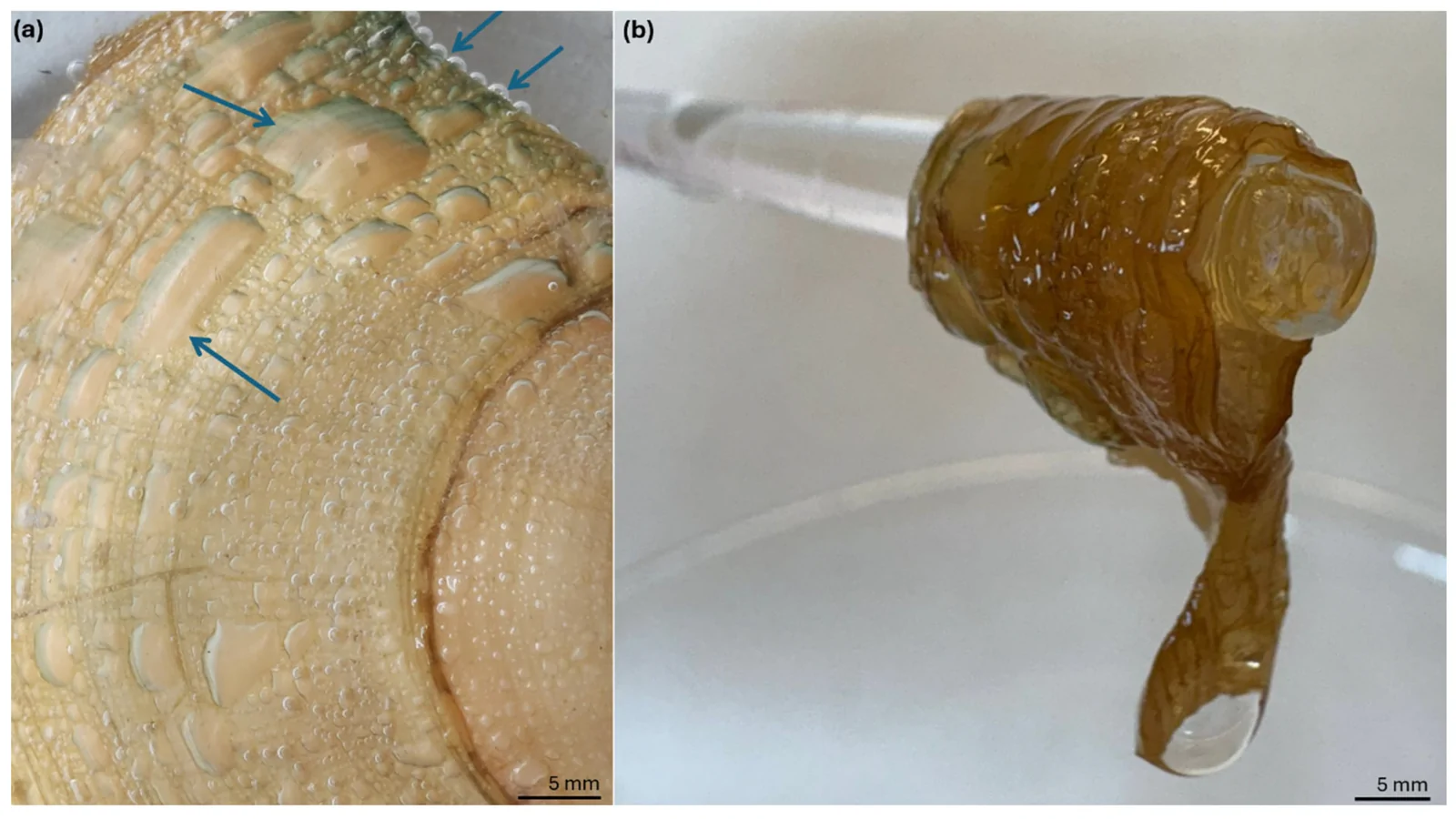
Decalcification of the shell of the freshwater bivalvian mollusk (**a**) using 15% HCL results in the intense release of CO_2_ (arrows). The conchix isolated from the shell under study is still elastic after 7 days of demineralization (**b**).

What happens if you carefully dissolve the mineralized mollusk shell in its original form, that is, without preliminary grinding, as was traditionally done before? Then we will get the so-called conchix, a biocomposite material consisting of structural proteins (i.e., keratin-like conchiolin), glycoproteins, chitin, pigments, and a lipid component. According to our definition, “*conchixes are a biocomposite material containing a e set of biomacromolecules, with the exception of water-soluble ones, that were originally present in shells*” [354,355] (Figure 5).

Conchixes possess one common feature: they completely repeat not only the contours of the shells but also their size, differing, however, in some species in the color of the originally pigmented organic phase (Figure 5b) [354,356,357]. Despite the fact that one of the main components of the matrix, conchoilin, was previously subjected to pyrolysis up to 900 °C (for details, see [339]), there is still no data on its use in either hydrothermal synthesis reactions or for carbonization in the scientific literature. It remains to be hoped that in the near future the conchixes from molluscan shells will find applications in the framework of extreme biomimetics as promising biological waste.

##### Fish Scales

Fish scales represent multi-layered natural dermal armor with remarkable puncture resistance and flexibility due to a well-balanced mix of organic components (guanine, collagen, lipids, lecithin, sclerotin, carotenoids, melanins, etc.) [358,359,360] and inorganic minerals (calcium phosphates; calcium-deficient hydroxyapatite), as well as trace elements such as iron, zinc, and magnesium [361]. Being an example of biowaste on a global scale [362], fish scales have been intensively investigated in recent times [363,364], and not only as a raw material for obtaining collagen [365], gelatine [366], or hydroxyapatite [367]. Numerous examples are excellently represented in the modern review by Dong and others entitled “*Fish Scales: A Multifunctional Biomaterial from Nature*” [368].

Fish scales are of importance for modern biomimetics in two ways: either as a model for inspiration (i.e., bio-mimicking fish scale textures) or as a biomaterial that can be chemically modified to produce a new composite for use in the relevant field. The first option is the most convenient due to the algorithm of the scientific approach, which consists of studying the features of the structural organization of fish scales using modern methods and creating corresponding biomimetic models for engineering imitation (i.e., structural biomimetics).

For example, structural design inspiration derived from fish scales led to the design of the layer-by-layer assembly of a novel biomimetic fish scale structure onto a carbon fiber surface via thiol–ene click chemistry [369] as well as of a bionic fish scale microchannel heat exchanger made by powder bed fusion additive manufacturing [370]. Liu and others reported on the creation of bioinspired magnesium composites with fish scale-like orthogonal plywood and double-Bouligand architectures using pressure-less infiltration of a magnesium melt into the woven contextures of continuous titanium fibers [371]. Creating a bioinspired fish scale structure with varying thickness using compression molding by sequentially grafting graphene oxide and carbon nanotubes onto the surface of carbon fibers via thiol–ene click chemistry also attracted attention [372]. Plate-like guanine biocrystals discovered on the surface of fish scales [359] found application in the development of diverse biophotonic biomimetic models (for details, see [373,374,375,376]).

In contrast to the inspirational examples above, the prospects for using fish scales themselves as templates for creating new composites are primarily limited by their potential contamination with heavy metals [377,378,379] or toxic chemicals of various origins. From the view of extreme biomimetics, observations of structural anomalies on fish scales due to accumulation of high, but not lethal, concentrations of heavy metals [380] could be of interest as possible examples of unusual biocomposites, for example, some kind of heavy metal-doped hydroxyapatite.

Remaining thematical, when analyzing the interaction of metal ions with fish scales, we would like to mention a number of published examples. Thus, the synthesis of CuO nanoparticles (CuO NPs) using fish scale waste under such calcination temperatures as 100 °C, 300 °C, 500 °C, and 700 °C has been reported previously [381]. In the case of the fish scale from commercially important Indian major carp *Labeo rohita* also used for the synthesis of CuO nanoparticles, it was observed that upon heat treatment, the collagen content of this fish scale got transformed into gelatin, which in turn converted the precursor material into CuO NPs [382]. In another study, carp scales modified with cerium oxide nanoparticles have been developed and proposed as a new bio-adsorbent for arsenic and chromium separation [383]. Fish scale-loaded TiO_2_ composites were prepared through the sol–gel method and investigated as photocatalysts in the degradation of Methyl Orange under solar light irradiation [384]. Also, monodispersed gold nanoparticles with enhanced dispersion stability for theragnostics have been produced using fish scale waste [385]. Silver-containing composites obtained due to the application of fish scales are known, too. Thus, Ag nanoparticle-embedded fish scales as SERS substrates for sensitive detection of corresponding chemicals have been recently reported [386]. Intriguingly, Murugan and others published data on bio-fabricated sardine fish scale silver nanoparticles used against the malarial vector *Anopheles stephensi* [387].

Similar to other aforementioned biopolymers and biocomposites, fish scales have not gone unnoticed as a substrate for producing metal-free composite materials, often using other natural raw materials or purely synthetic substances. For example, fish scale- and coconut shell powder-based composites have been developed and approved [388]. Fish scales were used as a bioadditive to enhance the thermal insulation and mechanical properties of coir–polypropylene composites [389]. A novel textile material, from upcycling of fish scales used for modification of polyester via a supramolecular method, has been manufactured and suggested to make a valuable contribution to the textile industry [390]. Fish scales have been applied as a sustainable reinforcement material together with maleic anhydride-modified acrylated epoxidized soybean oil as the matrix for green composite development [391]. Investigations on the biowaste fillers extracted from fish scales on the mechanical properties of high-density polyurethane foams have also been carried out [392].

The thermal properties of fish scales have implications for experimental approaches in extreme biomimetics, including the field of hydrothermal synthesis of composite materials. Interesting information can be found in the work entitled “*Thermal Behavior and Physicochemical Properties of Fish Scales for the Generation of Value–Added Products*” [393]. It is found that the composition and residues generated by the burning of fish scales can potentially be applied to soil conditioning, biomedicine, adsorbents, fertilization, and even asphalt production [393]. Furthermore, fish scales have been investigated as a natural flame retardant material [394]. Fish scale valorization by hydrothermal pretreatment is commonly used to enhance protein recovery without demineralization [395]. Thermal decomposition of chemically treated fish scales, originating from an Amazon fish species (*Arapaima gigas*) at different temperatures including 600 °C, 800 °C, and 1000 °C, led to the production of chlorapatite [396].

Of course, the carbonization of fish scales has not bypassed this biocomposite material (see overview, [397]). Carbon dots have been fabricated using readily available grass carp fish scales via one-step synthesis based on a pyrolytic reaction [398]. A hierarchical lamellar porous carbon material prepared from fish scales was proposed to be applicable for high-performance electrochemical capacitors [399]. Biochar derived via pyrolysis from fish scales has been used as a sustainable filler material in carbon fiber epoxy composites [400]. Carbonized fish scales possess the potential to be converted into photothermal materials such as carbon nanotubes (CNTs) or carbon nanodots (CNDs) for seawater desalination processes [401]. Also, self-nitrogen-doped carbonaceous nanostructured photocatalysts have been synthesized from fish scales by a hydrothermal method in the absence of any other reagents [402]. In addition, the preparation of graphite from fish scales has been reported, too [403].

It remains to be hoped that in the near future, such a uniquely hierarchically structured multiphase-based biocomposite as fish scales, briefly presented above, will find its niche in extreme biomimetics.

##### Spongin

What humanity has commonly called *a sponge* since the time of ancient Egypt is nothing more than the purified skeleton of a marine bath sponge from the order Dictyoceratida (Demospongiae), which consists of a biocomposite fibrous material called spongin [19,347] (Figure 3c). The microfibers themselves, 50 to 100 µm thick, consist of a central collagenous core [187] and a cuticular layer up to 700 micrometers thick of keratin-like origin [21]. The entire spongin fiber is cross-linked by various halogenated di- and tri-tyrosines, including iodine-, bromine-, and chloro-tyrosines, making it a chemically super-strong cross-linked biomaterial [187]. The spongin skeletons of sponges always have a 3D microporous structure, readily absorb a wide variety of liquids, and are extremely elastic (for details, see [19]). Furthermore, recently, we demonstrated the unique shape-memory behavior of spongin scaffolds even after two months of exposure to high mechanical pressure (20 mt per cm^2^) at temperatures exceeding 100 °C [19].

Being thermally stable in the presence of oxygen up to 360 °C, spongin is a promising biomaterial for the creation of 3D composites under hydrothermal synthesis reaction conditions (for overview, see [7,12,13,404]). It should be taken into account that despite the resistance of spongin to acids and organic solvents, in contrast to cellulose and chitin, it quickly undergoes decomposition and hydrolysis under alkaline conditions. At the same time, placing and maintaining spongin in an extremely aggressive chemical environment containing copper chlorides in an ammonia solution leads to the production of copper-containing composites with high catalytic activity (for details, see [9]). The copper-containing nanocrystalline phase is so firmly embedded in the spongin fiber that it remains there after sonication for several hours.

Thermostability of selected biomaterials is summarized in Table 2.

**Figure 6:**
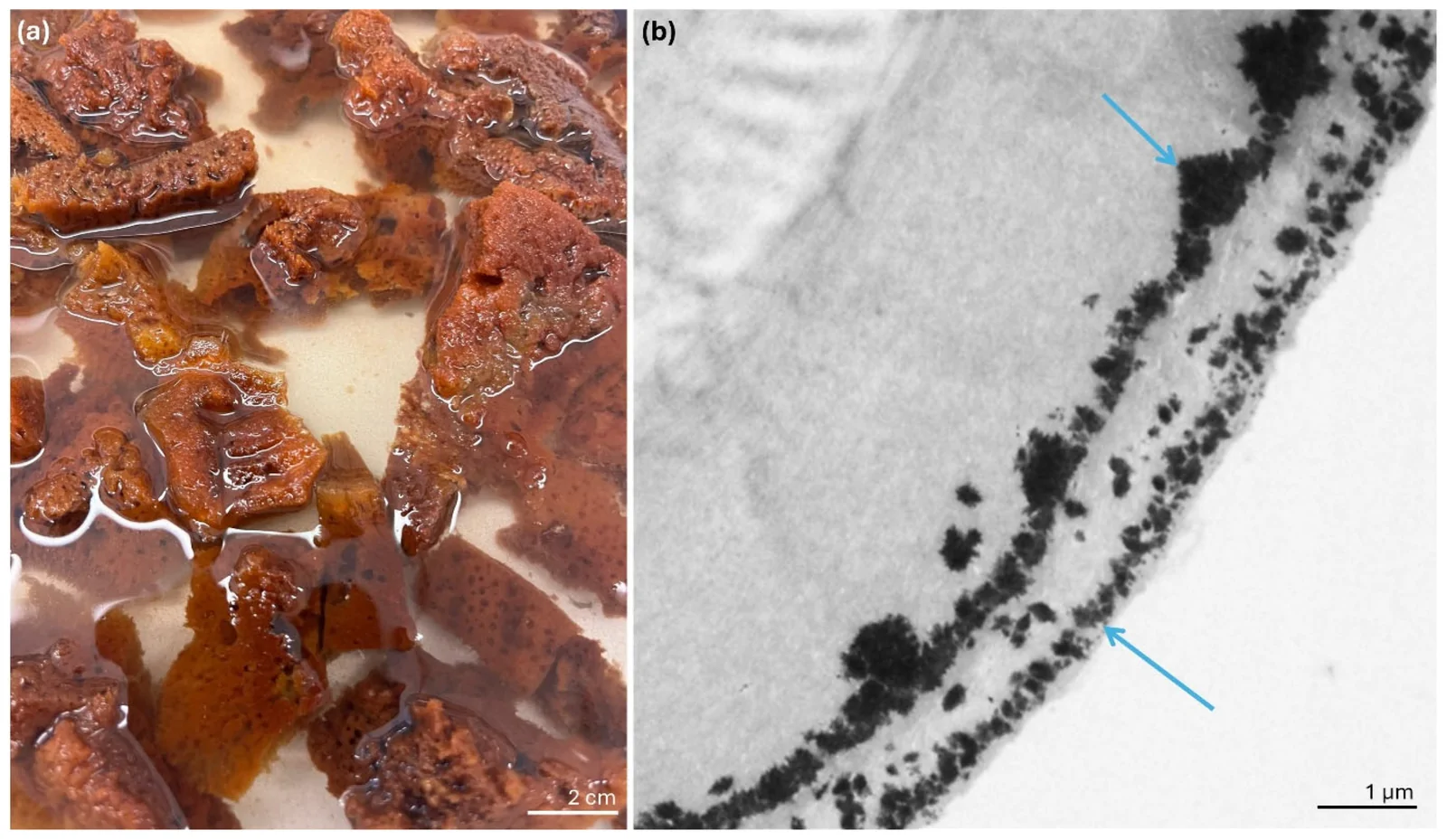
Fragments of the naturally occurring *Spongia officinalis* bath sponge collected near Bari (Italy) with a rusty color (**a**) due to the presence of iron oxides (i.e., lepidocrocite) tightly bound to and located within the 500 nm thick cuticular layer of the spongin fibers as represented on the TEM image ((**b**) arrows).

As a result of our long-term observations of spongin scaffolds obtained from the relevant commercial companies, where they undergo chemical processing in order to acquire an appearance and color acceptable in cosmetics, we have found a large number of so-called rusty sponges (Figure 6a) [14,15,19]. As it turns out, this color is formed due to dense nanostructured layers of iron oxides in the form of lepidocrocite and goethite, which formed on the spongin fibers as a result of the biocorrosion of iron structures in seawater and the formation of supersaturation of iron ions in localities around the sulfur-containing cuticle of the spongin fiber [14].

These types of rusty sponges are classified as waste in the production of cosmetic sponges and have abrasive properties rather than being harmful to the human body. However, we have proposed applications for them in various types of sensor materials (for details, see [340,405,406]). The next challenging task is the attempt to use rusty sponges as bio–iron–ore for melting under 1450 °C with the aim of obtaining steel-like composite materials.

**Figure 7:**
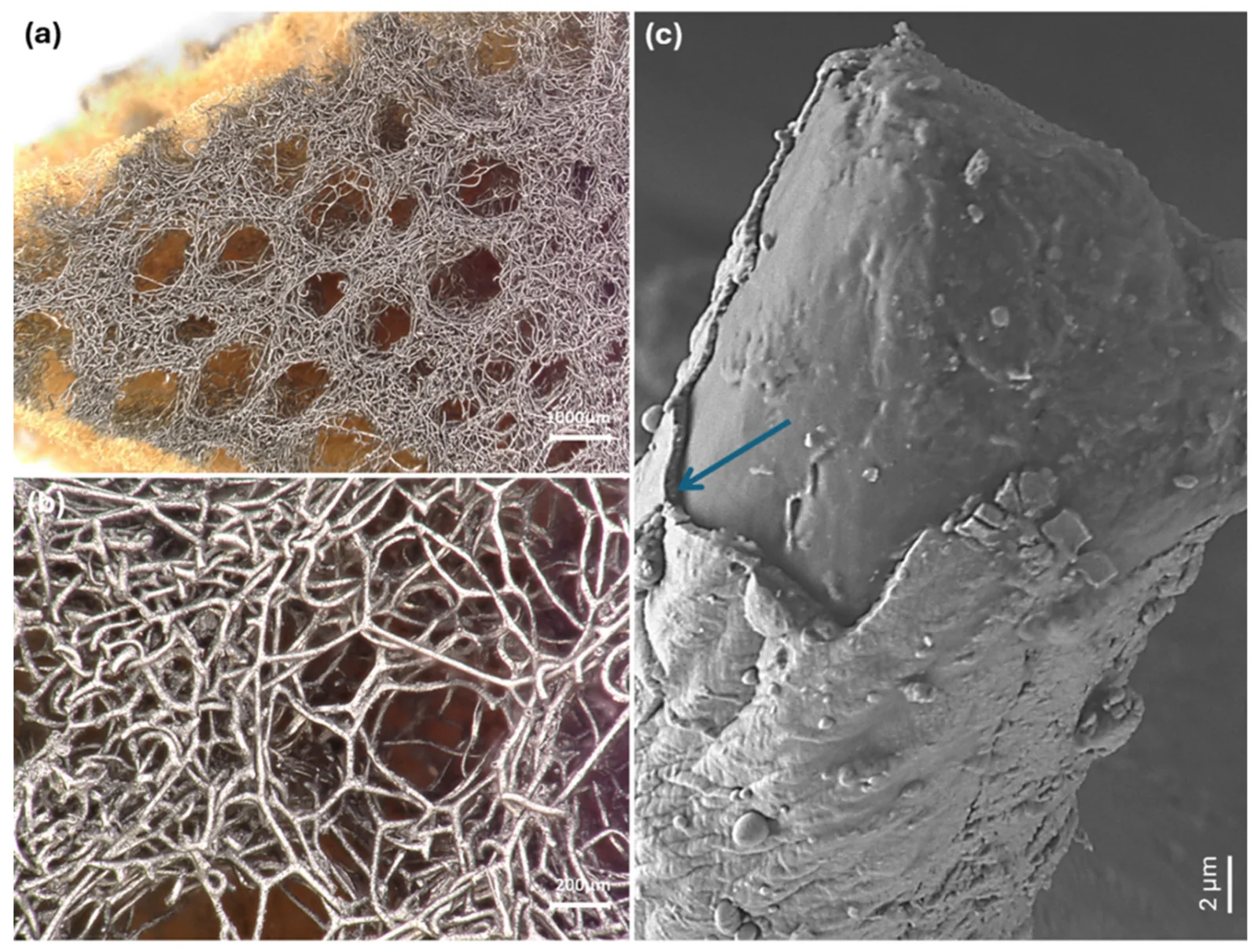
Ion-plasma (Vacuum Arc) deposition of titanium nanophase on the surface of spongin does not lead to a violation of the structural homogeneity of the spongin matrix, as exemplified by the scaffold of the *Spongia lamella* bath sponge (**a**,**b**). SEM image (**c**) represents the distribution of titanium micro- and nanoparticles on the cuticular 500 nm thick layer (arrow) of the spongin fiber.

The thermostability of spongin facilitated its coating with titanium nanoparticles at a temperature of about 300 °C without the loss of 3D structural integrity. Consequently, the creation of spongin–titanium 3D composites using the ion-plasma (Vacuum Arc) deposition method has been carried out successfully [19] (Figure 7).

A number of our studies are devoted to the carbonization of spongin [407], in which the transformation of carbon into turbostratic graphite is demonstrated, clearly recorded at temperatures from 1200 °C [20] to 2200 °C [21].

**Figure 8:**
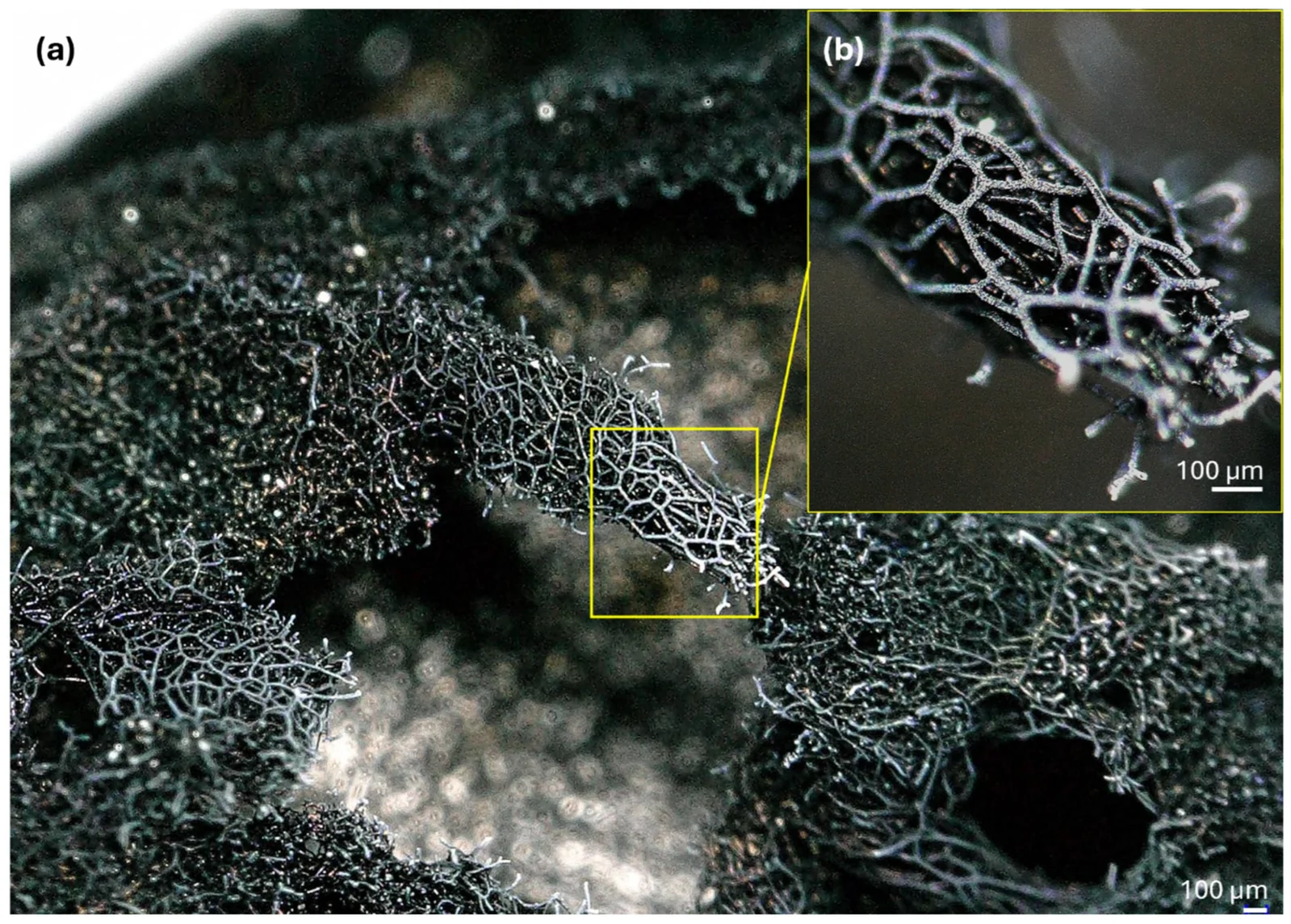
Digital microscopy image of 3D spongin scaffold obtained from *H. communis* bath sponge after carbonization at 2200 °C shows the typical sponge-like architecture that remains exceptionally preserved (**a**,**b**) despite the known losses in volume up to 70%.

One of the crucial features of carbonized spongin scaffolds is that even at temperatures up to 2200 °C, there is no loss of the structural integrity or sponge-like form (Figure 8), in contrast to other biomaterials, even those initially having a 3D structural organization (i.e., cellulose-based *Luffa* sp. plant sponge) (Figure 3a), which crumble into powder when carbonized at such temperatures [21].

At 2200 °C, the evolution of spongin graphitization led to the formation of turbostratic layered graphite with a surface containing diverse carbon nanophases including graphene, C60 nanocrystallites, and unique rhombohedral graphite with monoclinic *C*2/*m* space group symmetry [21].

**Figure 9:**
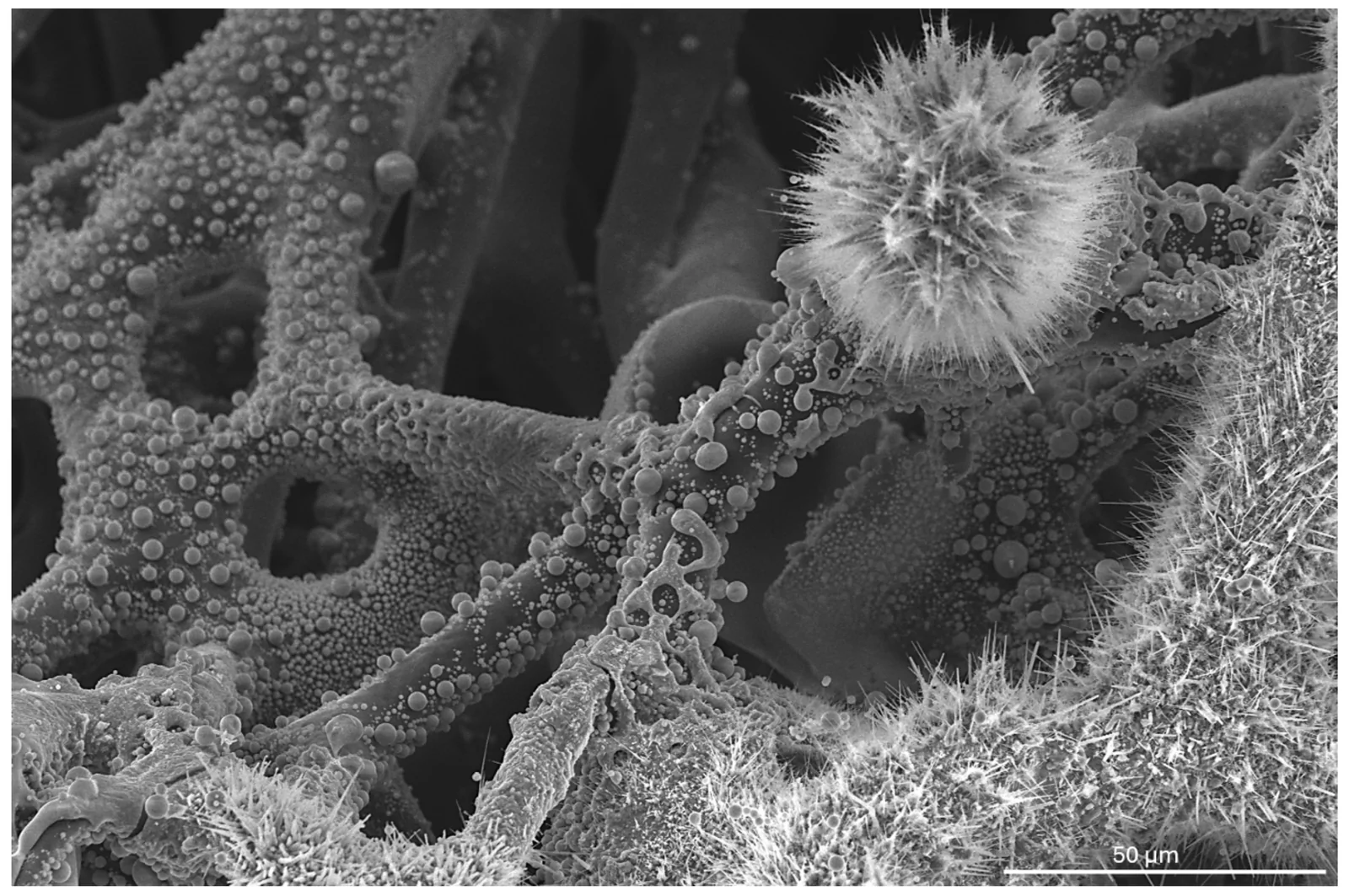
SEM image shows carbonized at 1200 °C spongin from *H. communis* demosponge not initially treated with 20%HF to dissolve silica. Consequently diverse spherical and needle-like microstructures confirmed as silica using EDX analysis turned out to be possible to observe.

The above facts apply to highly purified spongin, devoid of mineral components. However, in nature, this poriferan biocomposite often contains traces of silica- and aluminosilicate-based impurities. By carbonization of such natural silica-containing spongin, it is often possible to obtain previously unknown types of composites with outlandish nanoarchitecture (Figure 9). The future will show whether we can assume the formation of silicified turbostratic graphite with a 3D microarchitecture and as yet unknown functionality.

**Figure 10:**
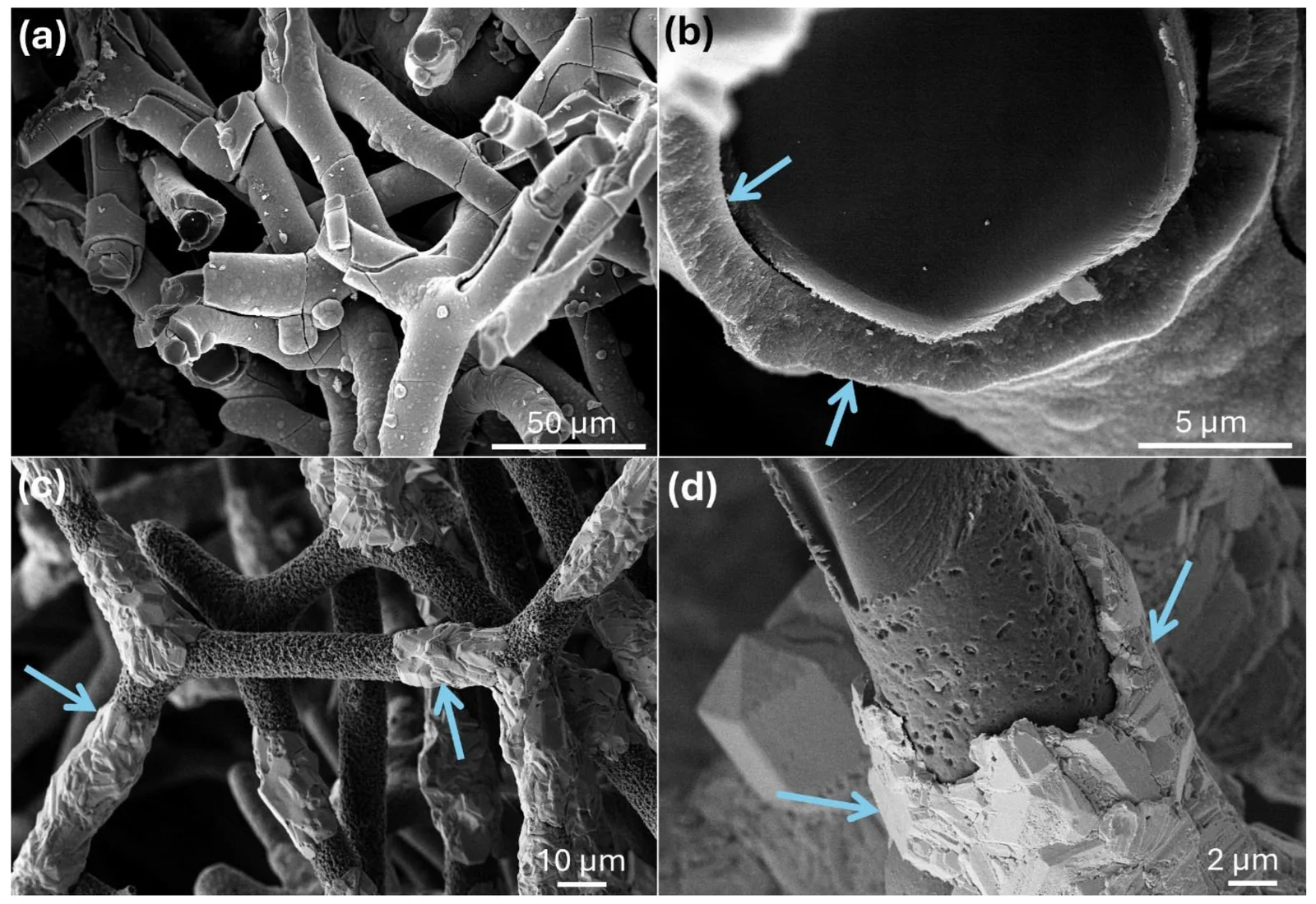
SEM imagery: carbonized spongin fibers of *H. communis* demosponge origin hydrothermally covered with a microlayer identified as MnO_2_ (**a**) remaining stable ((**b**) arrow) even after 1 h of ultrasonic treatment (for details, see [13]). Turbostratic graphite-based 3D scaffolds from the same sponge species can also be covered with up to 1 µm thick layers of copper oxides ((**c**,**d**) arrows) using electroplating.

Carbonized spongin can also be metallized using either hydrothermal synthesis or electroplating. In both cases, the surface of metal oxide-modified carbonized spongin remains highly resistant to ultrasonic treatment (Figure 10). Recently, it has been shown that steel melting at 1500 °C on 3D carbonized spongin scaffolds under extreme biomimetic conditions led to the development of so-called magnetic sponges [18]. Chromium tanning of spongin scaffolds isolated from two species of industrially used commercial sponges, *H. communis* and *Spongia tampa*, under extreme biomimetic conditions was recently reported by Lesniewski and others [17]. Carbonization of the studied 3D spongin scaffolds followed by chromium plating led to the formation of complex multiphase structures containing chromium carbides never reported before [17].

We would not hesitate to say that, due to the microplastic issue in the global environmental context, the era of synthetic sponges has come to an end. Natural spongin-based sponges, on the other hand, are experiencing a renaissance, especially due to their cultivation in marine ranching conditions. Also, a huge number of sponges remain lying in the warehouses of the corresponding companies due to sluggish demand from stores selling cosmetics [19]. Therefore, in our opinion, there is an urgent need to find new, besides cosmetic, applications for spongin matrices, namely in various advanced technologies from the adsorption of radionuclides to their use in the creation of new composite functional materials with unique 3D architectures that were approved by nature during more than 800 million years of evolution.

## 3. Scientifically Based but Daring Experimental Decisions in Extreme Biomimetics

Extreme biomimetics is a scientific discipline that offers unlimited freedom of action and choice of research topics to work on, although sometimes at one’s own risk. One of the key points is the fundamental ability of the biopolymers selected for research to withstand critical temperatures, pressure levels, and degrees of aggressiveness of the chemical environment without total destruction of their structural organization and dramatic changes in their physicochemical properties. Another important factor is the willingness and desire of colleagues who have the necessary specific equipment to conduct some illogical or, in their opinion, daring experiment.

Let us cite one actual example. In 2017, Mr. Iaroslav Petrenko, a graduate student, was tasked with placing a spongin scaffold in a special furnace at the Faculty of Metallurgy at the Technical University of Freiberg, Germany, with the goal of heating it to 1200 °C in an argon atmosphere. The metallurgists listened to his request with great caution and, just in case, inquired about the mental health of his supervisor, Professor Ehrlich. However, at the end of the negotiations, they finally agreed to the experiment, which ultimately led to the publication of the article in *Science Advances* entitled “*Extreme Biomimetics: Preservation of molecular detail in centimetre scale samples of biological meshes laid down by sponges*” [20] that is cited today 87 times. Mr. Petrenko successfully defended his doctoral dissertation in 2018 as the first PhD student in extreme biomimetics!

Our irresistible desire to test spongin in an extremely aggressive ammonia solution of copper chloride, used as a model solution to simulate real industrial waste from the processing of circuit boards for the production of electronics, was received with no less caution by colleagues from the Institute of Chemical Technology of the same university. This attempt culminated in the publication of an article in 2021 in *Advanced Materials* entitled “*Extreme Biomimetics: Designing of the first nanostructured 3D spongin–atacamite* composite *and its application*” that is cited today 45 times [9]. Therefore, when we melted steel on carbonized scaffolds obtained from marine sponges, or bombarded them with titanium nanoparticles under plasma arc conditions, no one was surprised, but rather expected some unusual and interesting scientific results. It is not surprising that extreme biomimetics, which arose at the intersection of such disciplines as paleomimetics [408], geomimetics [409], extremophile’s biomanufacturing [410], astrobiology [411], biomineralization [412], hydrothermal synthesis chemistry [413], solid state physics [414], biomimetic additive manufacturing [415], and bioinspired materials science [274] (Figure 1) has today become multidisciplinary and automatically presupposes mutual understanding between scientists of various fields, especially those who have not lost their purely human interest in pioneering and discovering.

### 3.1. Extreme Carbonization of Naturally Pre-Structured Biomaterials

Carbonization of biological materials, either to produce various forms of activated carbon or to graphitize 3D skeletal scaffolds already pre-structured by corresponding organisms during their evolution, is logical for the purposes of extreme biomimetics. One of the intriguing questions regarding the carbonization of such matrices is the maximum temperature limit at which the organic matrix, when carbonized, will retain its original architecture. Until recently, the world temperature record for the carbonization of biomaterials concerning the transformation of silk from the cocoon of the silkworm into so-called pseudographitic crystalline pyroproteins has been registered at a temperature of 2800 °C [21,416]. Surprisingly, one of the main limitations for graphitizing biomaterials at higher temperatures is the lack of appropriate equipment. For example, metallurgical applications require specialized furnaces capable of reaching temperatures of up to 1600 °C. Kilns with a capacity of up to 2500 °C can be used for the production of some ceramics.

**Figure 11:**
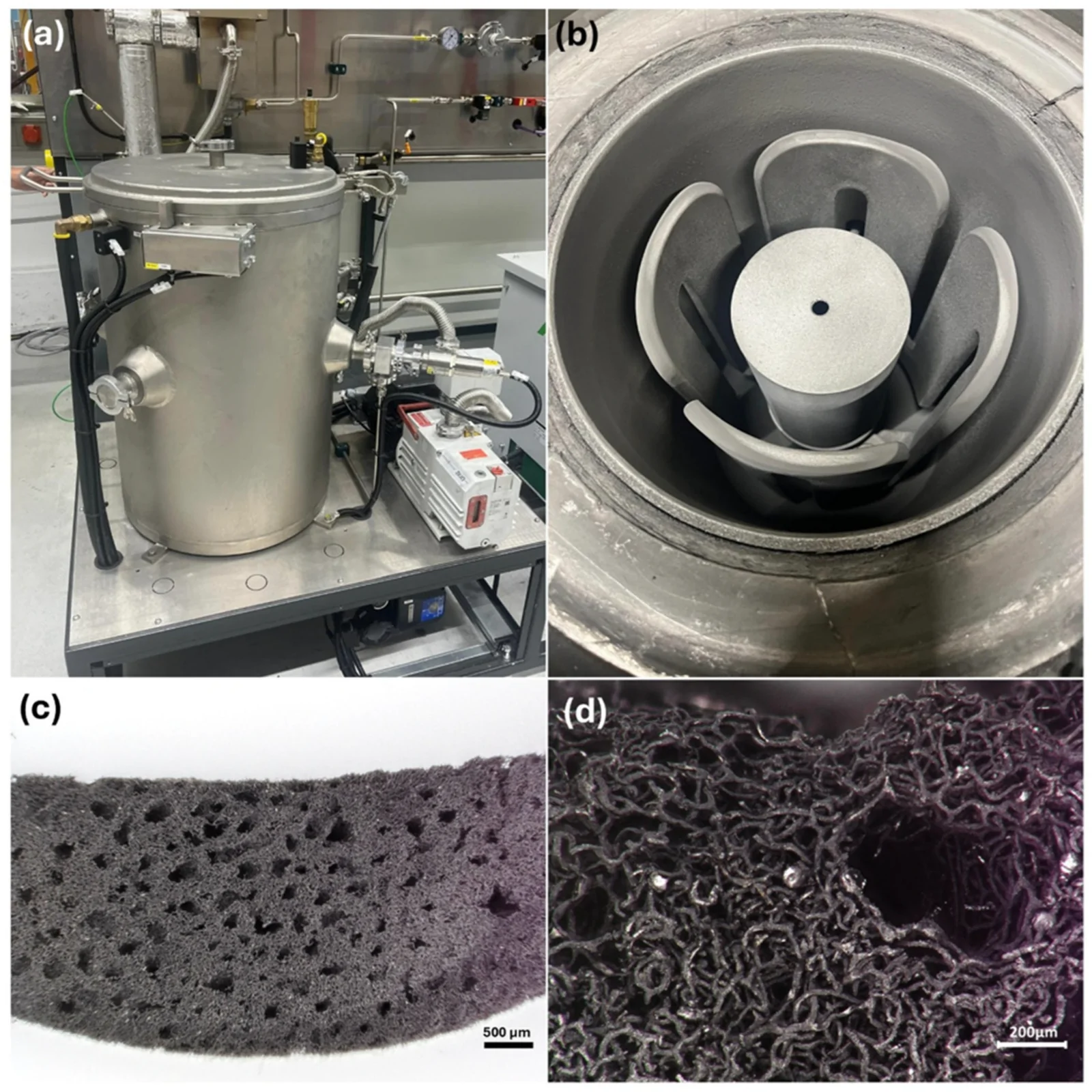
Highly specialized furnace (**a**,**b**) used for carbonization of ordered spongin scaffold initially covered with Ti under plasma arc conditions. Digital microscopy images of this spongin scaffold after carbonization at 3000 °C (**c**,**d**) confirm the exceptional preservation of its microarchitecture and natural 3D design.

After we recently published very interesting results on the carbonization of spongin at 2200 °C [21], we set ourselves the ambitious goal of carbonizing spongin at 3000 °C. But where can we obtain such equipment? By good fortune, we were able to conduct the relevant experiments using specialized furnace equipment from Carbolite Gero GmbH & Co. KG in Neuhausen (Germany) (Figure 11), which allowed us to record a temperature of 3000 °C during the carbonization of a 3D spongin scaffold isolated from *S. lamella* marine demosponge initially modified with titanium nanoparticles using the plasma arc method [19]. This experiment demonstrated the unique preservation of the 3D architecture of titanium-coated carbonized spongin at such a high temperature. We are currently conducting comprehensive analytical studies to identify the resulting carbon phases and, possibly, titanium carbides. It is known that TiC can be formed by the reaction of solid carbon and titanium dioxide at a high temperature up to 2000 °C and a long reaction time of up to 20 h [417]. But what kind of product was created at 3000 °C remains a matter of speculation.

It should not be forgotten that this success was preceded by many years of research since 2017 on the carbonization of spongin as a biocomposite-based natural material with sophisticated nanoarchitectonics at temperatures from 300 °C to 2200 °C [13,20,21,418,419]. Now, after reaching a carbonization temperature of 3000 °C for biomaterials using spongin as an example, new research prospects are opening up, perhaps more daring than our successful attempt to disarm pessimistic colleagues from various scientific disciplines.

**Figure 12:**
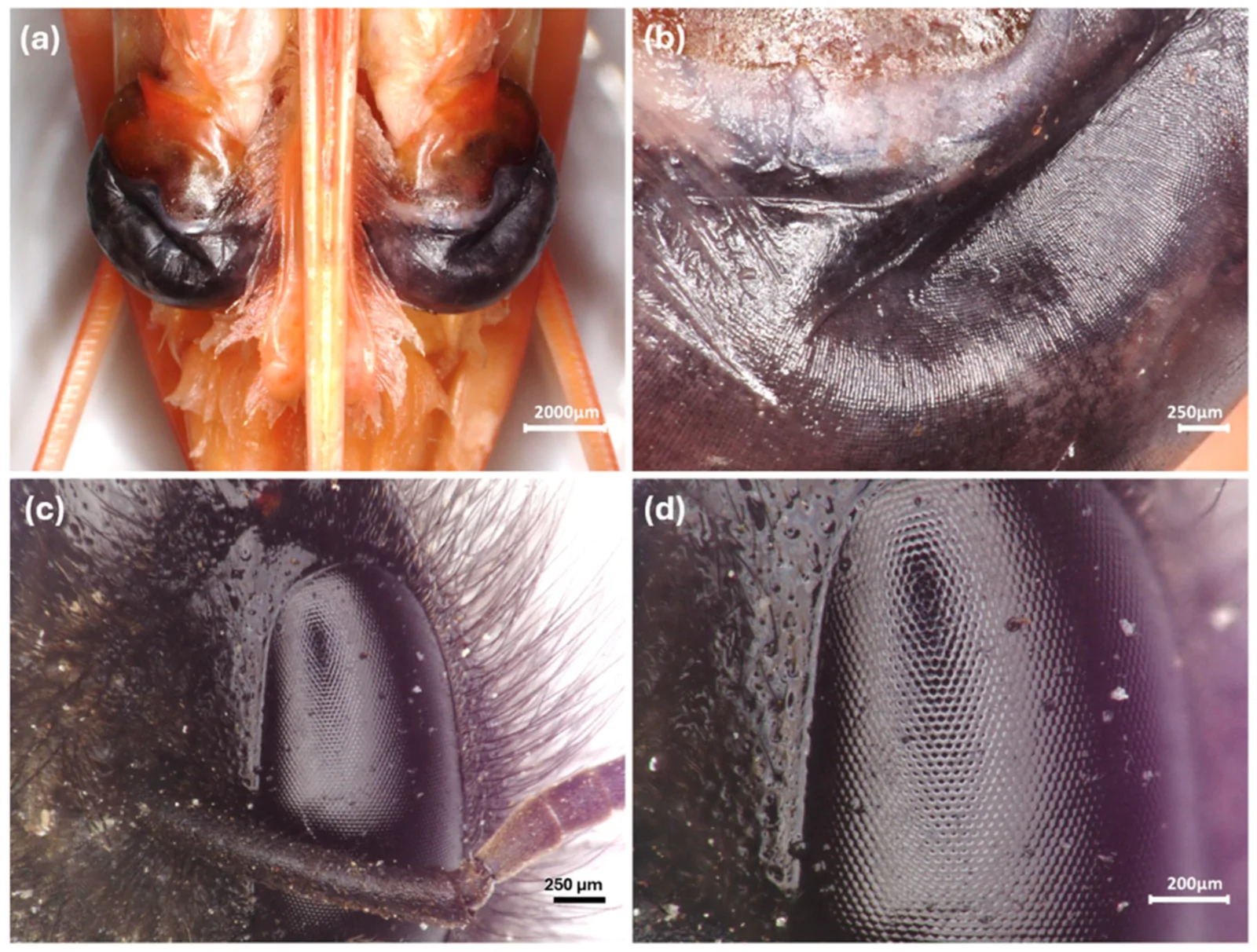
Digital microscopy images represent characteristic hexagonal structural motifs of the pigmented with melanin, chitinous compound eyes observable in krill (**a**,**b**) and in bees (**c**,**d**).

As another example of pre-structured biopolymer-based matrices that could potentially be decorated with metals and finally carbonized, we would like to suggest the compound eyes of insects and crustaceans with characteristic hexagonal microarchitecture (Figure 12) [419,420,421]. A compound eye represents a visual organ found in both insects and crustaceans that consists of thousands of bio-optical units known as ommatidia, with a typically hexagonal architecture well visible in cross-section. Such a hexagonal outer surface of the compound eye acts as the primary lens to gather and focus light [420], as well as contributing to camouflage by reducing glare to predators [419]. Equidistant connectivity, a lesser quantization defect, and greater angular resolution belong to advantages of the hexagonal eyes [422,423].

Traditionally, it is the architectural features of the arthropod eye structure that are actively used as bio-inspired biomimetic models for the creation of artificial photonic systems using diverse advanced technological bio-replication approaches of compound eyes to create nanophotonic sensors and photovoltaics [147,424,425]. Recently, Takemura and others used low-temperature magnetron sputtering to directly replicate the compound eye structure of the dragonfly (*Pantala flavescens*) onto thin metal films using thickening of the thin Au-, Pt-, or W-containing film into a low-stress film, which aided in preserving the dragonfly molds intact for molding [426].

The ability to metallize the compound eyes of these invertebrates is due to the macrobiomolecules they are composed of. These are primarily nanofibrillar chitin and, secondarily, melanin. For example, the chitin content of the corneal lenses of the dragonfly (*Sympetrum fonscolombii*) was determined to be quite high (20.3 ± 0.85%) [427].

The outermost ommatidial pigment shield in compound eyes contains eumelanin, the same pigment that is found in human eyes [428]. Both chitin and melanin can react with a broad diversity of metal ions. Interaction of melanin with metals is represented and discussed in several papers (see for overview, [429,430]). Accordingly, our strategy consists of the initial metallization of the eyes of certain common arthropod species, which, after consumption (shrimp) or natural mass death (bees, locusts), become biowaste. After this, metallized faceted bio-scaffolds with a hexagonal microarchitecture can be carbonized under experimentally selected temperature conditions in order to obtain structured replicas of metal carbides. The question of the practical application of such functionalized compound eyes of insects and crustaceans remains open in its specifics, but is definitely solvable by interested researchers.

### 3.2. Galvanobiomimetics

Corrosion is an oxidation–reduction reaction occurring at the phase boundary. In this case, a transition from a metallic to an ionic state occurs. From the data presented in the Standard Electrochemical Series [431], it is easy to determine the incompatibility of metals and to assume the conditions under which so-called galvanic corrosion will definitely occur. In a galvanic series, a more noble metal acts as a cathode, and a less noble metal acts as an anode, dissolving. The phenomenon of galvanic corrosion is well investigated and mechanisms are proposed for diverse galvanic series (for details, see [432,433,434]).

**Figure 13:**
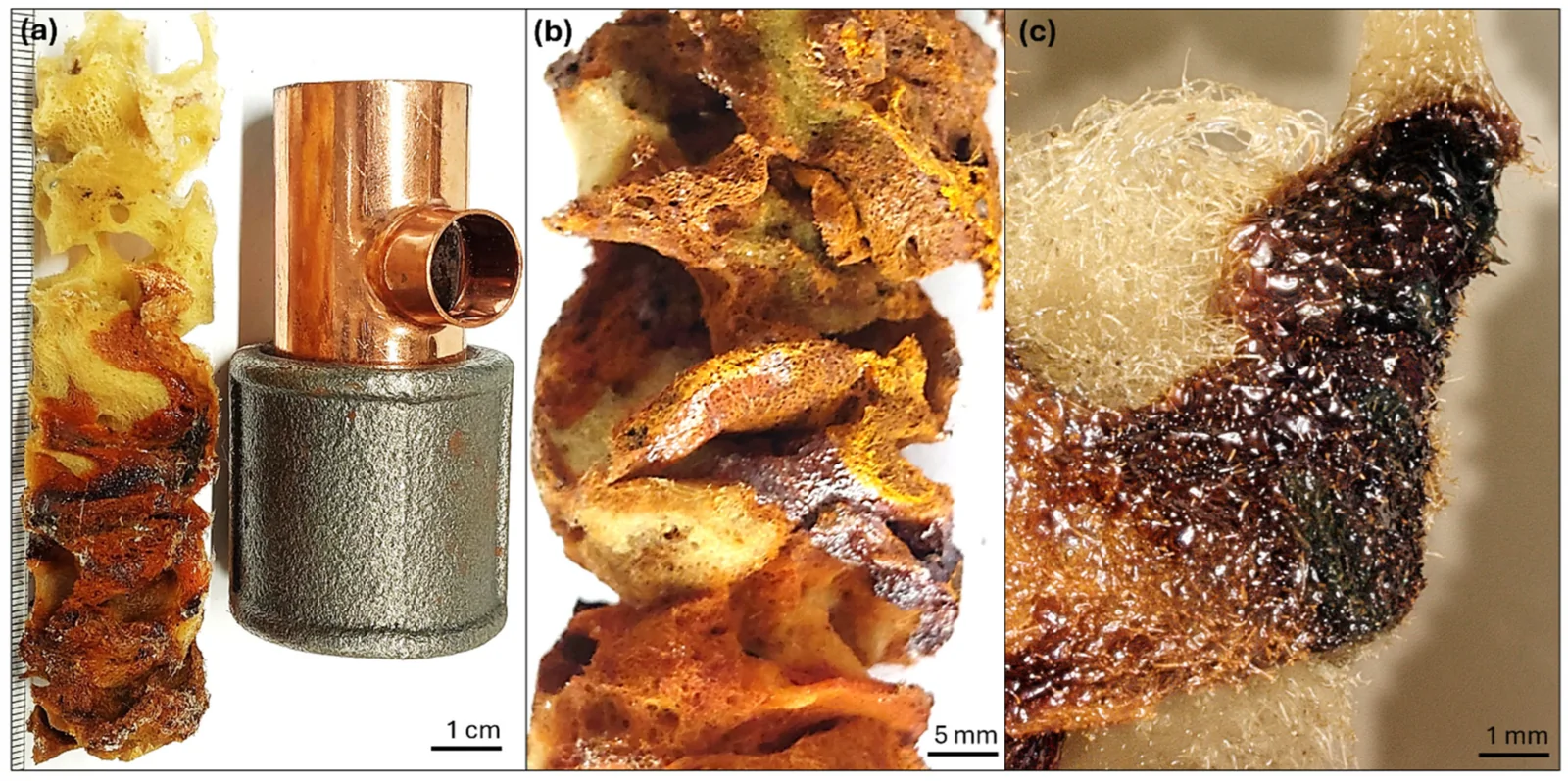
Construction used for the galvano-corrosion experiment. Photograph of the copper/steel setup (**a**) used to provoke the galvanic corrosion in the presence of a moistened with HCl spongin scaffold, which became rusty–greenish during the time (**a**,**b**). A digital microscopy image (**c**) representing the spongin scaffold location with intensive coloration typical for iron oxide phases.

Based on the logical and objective requirements for technological needs for joining parts made of metals of different origins, the main task is to avoid creating conditions for the occurrence of galvanic corrosion in principle. However, in extreme biomimetics, we are alternatively looking for methods useful to modeling galvanic situations with the aim of corrosion intensification. Why? To expose a 3D biopolymer matrix, such as a spongin scaffold, to such corrosive conditions and attempt to metallize it, awaiting the formation of the corresponding composite material. The first results are represented in Figure 13.

Thus, 3D spongin from an *H. communis* commercial bath sponge was initially moistened with a 10% HCl solution and placed into a copper/steel pipe construction, as presented in Figure 13a, where it was subjected to galvanic corrosion. The spongin sample placed inside the pipes was kept moistened by adding 10% HCl solution every two days for a period of one month.

It remains to be assumed that by selecting certain galvanic series and creating from them certain structures in the form of cells, inside it will be possible to place a spongin matrix, and as a result, metal oxides participating in the experiment will form or even crystallize on the surface of the spongin fibers, being tightly bound to them. Depending on the task at hand, appropriate scaffolds (Figure 3) made of cellulose, chitin, or keratin can also be used as templates. Also, the intensification of galvanic corrosion in the temporal aspect will be carried out in the near future as part of the strategy of extreme biomimetic experiments.

### 3.3. In Flame Biomimetics

Biopolymers, similar to diverse organic materials, are easy to ignite and consequently flammable [435]. At the same time, the behavior of various biomacromolecules as flame retardants, including chitosan, tannic acid, phytic acid, and lignin, has been studied quite intensively until recently [436,437,438,439]. It still remains important to improve the reaction of biocomposites to fire [438]. For example, a highly efficient flame retardant strategy using biomaterials with respect to biomimetics has already been established [439,440,441]. Fang and others proposed an eco-friendly approach to fabricate fire-resistant cellulosic Chinese Xuan paper by layer-by-layer assembly of mineral montmorillonite and chitosan [440].

**Figure 14:**
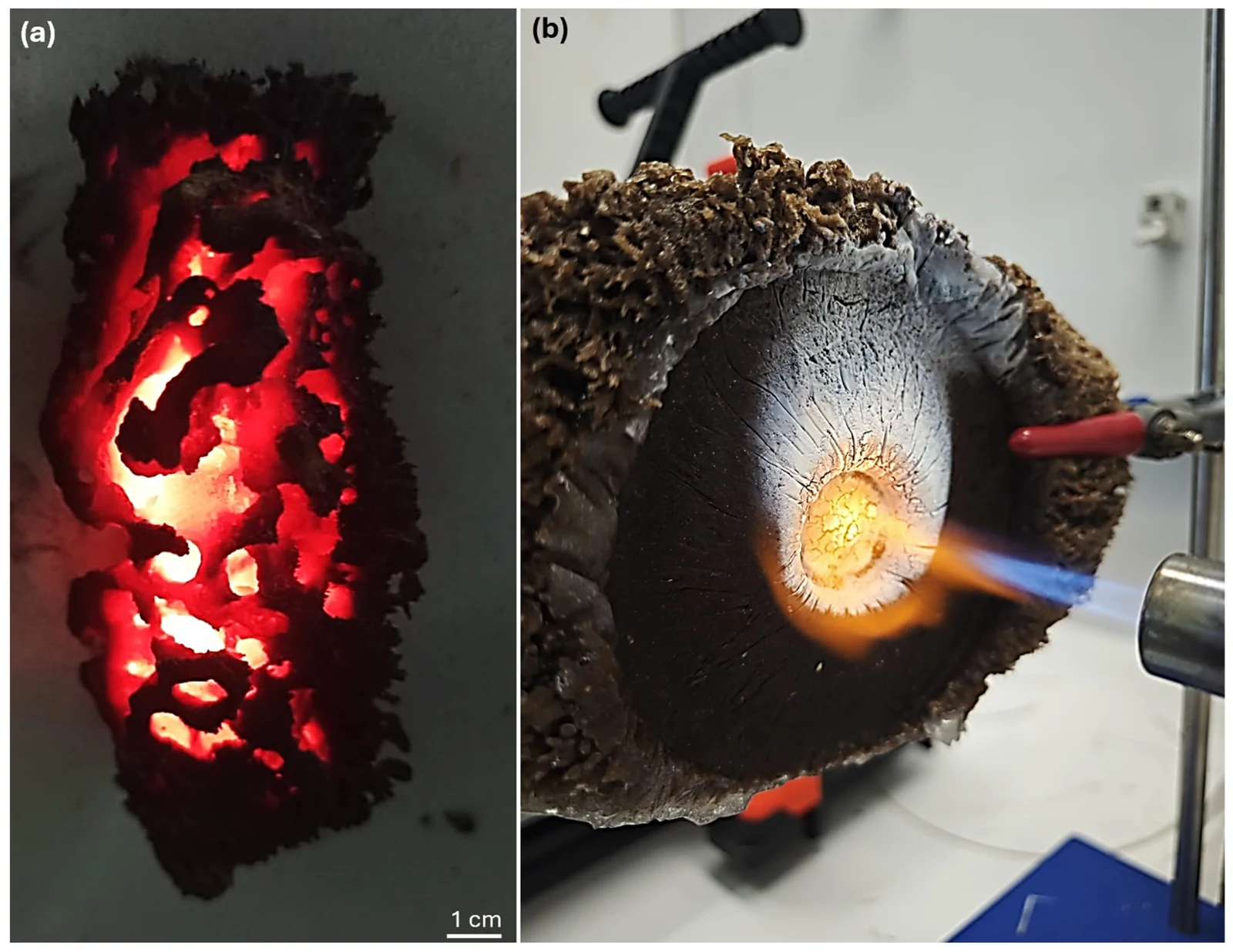
When ignited with a gas torch, the *H. communis* spongin matrix (**a**) exhibits surprising fire resistance. Even greater fire resistance is achieved with a spongin scaffold soaked in a silicone solution (**b**).

Naturally, we could not ignore the spongin scaffold in this regard, so we set it on fire. However, it burned strangely, not turning into charcoal (see Figure 14).

We believe that the observed effect is due to the high content of bromine and iodine in spongin [19,346]. Intriguingly, conventional halogen-based flame retardants are progressively restricted due to their toxicity. In the case of spongin, we obtained a halogen-based flame bioretardant with until today unknown properties, which will be investigated without doubt as soon as possible.

At the same time, we do not exclude that this kind of flaming spongin scaffold can also be considered a potential reactor in which combustion synthesis reactions [442] can be carried out. It is recognized that various nanoscale materials, including oxides, sulfides, nitrides, metals, and their alloys as well as magnetic particles, can be obtained using combustion synthesis [443]. Definitely, some role will be played by the keratinous component within spongin fibers. It was reported that thermal degradation of such keratin sources as sheep wool, human hair, and chicken feathers led to the formation of sulfur-containing inorganic compounds (SCS, SCO, H_2_S, and SO_2_ at 240, 248, 255, and 253–260 °C, respectively) [444]. Without a doubt, this new direction in extreme biomimetics, which currently contains more questions than answers regarding what final materials will be obtained from the combustion of spongin, will continue to develop despite the diversity of the challenging tasks.

### 3.4. Pyro-Biomimetic

Perhaps one of the rather daring approaches within the extreme biomimetics framework is our recent attempt to carry out, together with Prof. Robert Przekop, the famous Nicolas Lemery “*volcano experiment*” using a spongin scaffold and ammonium dichromate (IV). This experiment also has another name, “*Ammonium dichromate volcano*” [445], and demonstrates the following reaction of the thermal decomposition of this compound [446]:

(NH_4_)_2_Cr_2_O_7_ → Cr_2_O_3_ + N_2_ + 4H_2_O

During this reaction, orange-colored crystals of ammonium dichromate are ignited to erupt, and in this case, a violent release of steam and nitrogen gas occurs, and a large “mountain” of fluffy green chromium(III) oxide becomes observable.

According to Mahieu and others, the thermal decomposition of ammonium dichromate has the following reaction sequence: ammonium dichromate, a non-stoichiometric compound of Cr(V), chromium dioxide, and dichromium trioxide [447]. Recently, the temperature effect, mechanism, and kinetics of formation of Cr_2_O_3_ from (NH_4_)_2_Cr_2_O_7_ based on this thermal decomposition reaction have been reported, too [448].

**Figure 15:**
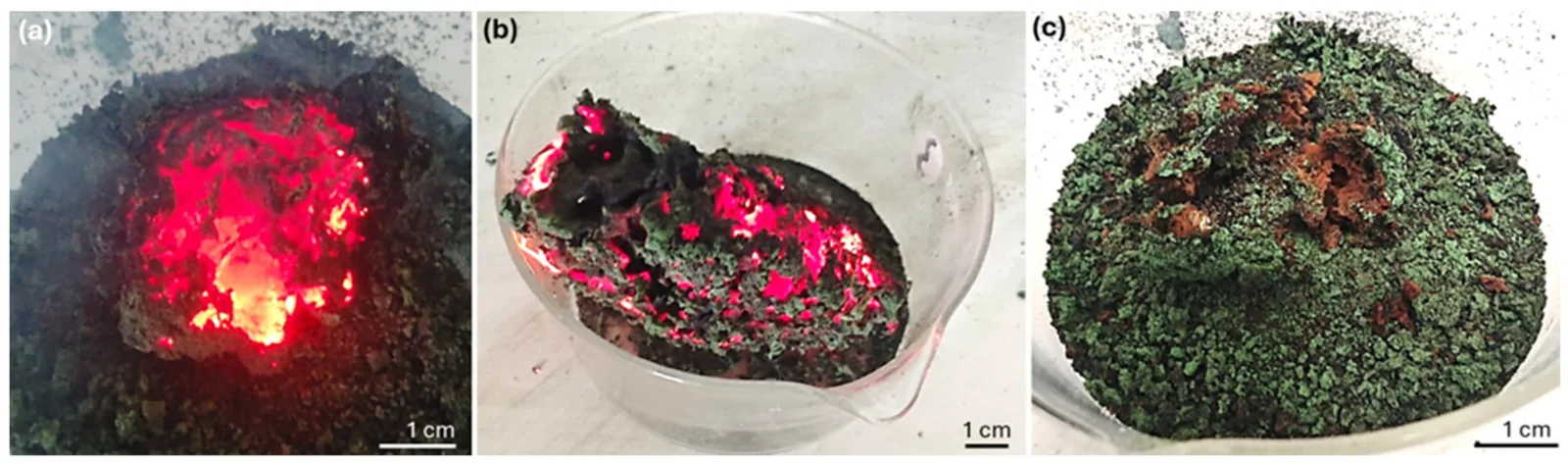
The situation inside the burning *H. communis* 3D spongin scaffold, saturated with ammonium dichromate, reminiscent of the mouth of a fire-breathing “volcano”, is clearly presented in the first picture (**a**). After the reaction is complete, residual fire spots continue to be observed in the cooling sponge (**b**), which has not lost its shape and size. The cooled sponge becomes coated with typically green chromium oxide particles (**c**), which become tightly bound to the 3D spongin matrix in which they formed as a result of the reaction described.

What happens if we saturate a spongin scaffold with ammonium dichromate and set it on fire? The result of the experiment, in which a temperature of 190 °C is achieved, clearly shows that despite the “volcanic-like activity” and all kinds of particle eruptions, the structure of the sponge is preserved (Figure 15). The unique ability of the spongin biomaterial to “survive” in such a chemically aggressive environment while maintaining its architecture never ceases to amaze.

So, we are currently continuing to intensively study what we have obtained. It is quite possible that we have a completely new composite material; the formation mechanism and its properties still need to be studied in detail to predict its practical applications. We have named this first example as one of the new directions in extreme biomimetics *pyro-biomimetics* and consciously believe that, using the same principle, it is possible to carry out other pyrotechnic reactions (i.e., Mg and Al compositions [449] or metal-based nanothermites [450]) already known to science [451,452], but using 3D matrices made of thermostable biomaterials other than spongin.

### 3.5. UHP (Ultra-High Pressure) Mimetic

The deep-sea habitat, as exemplified by the Mariana Trench with a pressure of 8 tons psi, has clearly attracted the attention of the scientific community, both in terms of biodiversity and the metabolic characteristics of extreme deep-sea organisms, including biomineralizing invertebrates and some fish species. It was reported that trimethylamine N-oxide found in the cells of deep-sea inhabitants is able to produce a protective effect against high external pressure [453]. Of particular interest for extreme biomimetics are the principles of biomineralization of skeletal formations in polychaetes and bivalvian mollusks recently discovered and described at depths of about 10 km [454]. One of the challenging tasks for future research is to understand the possible role of trimethylamine N-oxide on biomineralization under high-pressure conditions. In addition, it is worth noting that the development of bioinspired soft robots for deep-sea exploration [455] may draw inspiration in part from the architectural solutions of the skeletal structure and the locomotion features of deep-sea animals.

Large volume presses are widely investigated in geo- and planetary sciences with respect to the minerals and materials related to the deep Earth’s interiors (for details, see [456]). To mimic such natural conditions, special laser-heated diamond anvil cells to carry out experiments with ultra-high pressure up to the megabar regime [457] have been developed in the group of Professors Leonid and Natalia Dubrovinsky (University of Bayreuth, Germany) [458]. During the last decade, numerous materials have been formed under high pressure, including iron oxides with an unusual stoichiometry [456].

It is logical to assume that for extreme biomimetics, the use of such technological approaches to obtain completely new materials based on, for example, metal-modified spongin scaffolds graphitized at 3000 °C would be a unique and completely new experience. Of course, we made a corresponding proposal to Professor Dubrovinsky and, of course, received a quick refusal due to being busy with projects in a different direction. However, our idea to conduct this kind of experiment on the appropriate unique equipment will continue to exist until this event takes place.

## 4. Conclusions

Our review has included the historical moments of the emergence and development of extreme biomimetics in the last 10 years, as a scientific field that not only emerged at the intersection of various disciplines, but also continues to exist there today. The vast number of organisms living in extreme natural conditions for humans, on the one hand, and the peculiarities of the formation and structure of their skeletons, on the other, will continue to be the focus of attention for experts involved in extreme biomimetics. And what surprises nature will bring us after encounters with man-made disasters and spreading military cataclysms, one can only guess. Traditionally, we consider the doubts and skepticism of our scientific colleagues as one of the driving forces behind the evolution of extreme biomimetics. Green technology advocates ask what they see as a logical question about the advisability of testing biomaterials under sometimes critical physical and chemical conditions or on specialized equipment developed for metallurgy or the synthesis of materials under megabar pressure. First, extreme biomimetics utilizes already existing specialized and sometimes very expensive equipment of this kind, but for its own purposes. Second, freethinking in science, inventiveness, and a healthy, albeit sometimes ambitious, optimism in the scientific community have not yet been abolished. We also believe that the integration of artificial intelligence into the creation of composites inspired by extreme biomimetics and sometimes unique approaches will offer the generation of new biocompatible, sustainable, and sophisticated materials and corresponding technologies.

## Figures and Tables

**Table 1:** Selected properties of biological materials discovered in extremophilic invertebrates.

Extremophile	Biological Material	Physical Properties	Reference
Scaly foot snail *Chrysomallon squamiferum*	Conchiolin	Resistance to high sulfide concentrations and temperatures	[44,45]
Vent worm *Riftia pachyptila*	Chitin–protein composite	Thermostability	[55,56]
Pompeii worm *Alvinella pompejana*	Chitin; collagen	Thermostability	[58,59]
Pompeii worm *A. pompejana*	Chitin-protein composite	Conversion of excess heat	[61]
Pompeii worm *A. pompejana*	S-metals-based spherocrystals	Regulation of sulfur	[62]
Sulfide worm *Paralvinella sulfincola*	Mucous polysaccharides	Resistance to high sulfide concentrations and temperatures	[63,64]
Sulfide worm *Paralvinella hessleri*	Epithelial cell granules	Resistance to high As and H_2_S concentrations	[54]
Annelid *Pontoscolex corethrurus*	Chitin–protein composite	Thermostability; resistance to high metal concentration	[66]
Blind vent shrimp *Rimicaris exoculata*	Bacteriophore setae; pancreas granules	Bioaccumulation of Cu, Cd, and S	[70]
Hydrothermal vent barnacle *Vulcanolepas osheai*	Biocalcytic plates	Thermostability; resistance to high metal concentration	[76,77,78]
Shore flies *Ephydra* sp. larvae	Chitin–protein composite	Resistance to high Fe, Si, and mirabilite concentration	Personal observations

**Table 2:** Thermostability of selected biomaterials.

Biomaterial	Temperature of Degradation				References
	1st Stage	2nd Stage	3rd Stage	4th Stage	
Byssus	up to 200 °C	230–320 °C	above 30 °C	550–600 °C	[231,338]
Cellulose	below 100 °C (in nitrogen and air)	400 °C (in nitrogen) and 360 °C (in air)	475 °C in air	—	[116,117,118,128,338]
Chitin	50–140 °C	235–510 °C	—	—	[149,153,168,338]
Collagen (native)	111 °C	308 °C	570 °C	—	[209,210,338]
Conchiolin	pyrolyzed up to 900 °C				[338,339]
Keratin	below 200 °C	200–400 °C	410–650 °C	—	[338,339]
Silk	below 100 °C	310–480 °C	500–650 °C	—	[242,247,253,275,338]
Spongin	80–150 °C	200–420 °C	—	—	[7,12,13,338,404]

## Data Availability

No new data were created or analyzed in this study. Data sharing is not applicable to this article.
